# Inhibition of aberrant Hif1α activation delays intervertebral disc degeneration in adult mice

**DOI:** 10.1038/s41413-021-00165-x

**Published:** 2022-01-05

**Authors:** Zuqiang Wang, Hangang Chen, Qiaoyan Tan, Junlan Huang, Siru Zhou, Fengtao Luo, Dali Zhang, Jing Yang, Can Li, Bo Chen, Xianding Sun, Liang Kuang, Wanling Jiang, Zhenhong Ni, Quan Wang, Shuai Chen, Xiaolan Du, Di Chen, Chuxia Deng, Liangjun Yin, Lin Chen, Yangli Xie

**Affiliations:** 1 Center of Bone Metabolism and Repair, Department of Wound Repair and Rehabilitation Medicine, State Key Laboratory of Trauma, Burns and Combined Injury, Trauma Center, Research Institute of Surgery, Daping Hospital, Army Medical University, Chongqing, China; 2 Senior Department of Orthopedics, the Fourth Medical Center of PLA General Hospital, Beijing, China; 3 Department of Orthopedic Surgery, The Second Affiliated Hospital, Chongqing Medical University, Chongqing, China; 4 Research Center for Human Tissues and Organs Degeneration, Shenzhen Institutes of Advanced Technology, Chinese Academy of Sciences, Shenzhen, China; 5 Faculty of Health Sciences, University of Macau, Macau SAR, China

**Keywords:** Pathogenesis, Homeostasis

## Abstract

The intervertebral disc (IVD) is the largest avascular tissue. Hypoxia-inducible factors (HIFs) play essential roles in regulating cellular adaptation in the IVD under physiological conditions. Disc degeneration disease (DDD) is one of the leading causes of disability, and current therapies are ineffective. This study sought to explore the role of HIFs in DDD pathogenesis in mice. The findings of this study showed that among HIF family members, Hif1α was significantly upregulated in cartilaginous endplate (EP) and annulus fibrosus (AF) tissues from human DDD patients and two mouse models of DDD compared with controls. Conditional deletion of the E3 ubiquitin ligase *Vhl* in EP and AF tissues of adult mice resulted in upregulated Hif1α expression and age-dependent IVD degeneration. Aberrant Hif1α activation enhanced glycolytic metabolism and suppressed mitochondrial function. On the other hand, genetic ablation of the *Hif1α* gene delayed DDD pathogenesis in *Vhl*-deficient mice. Administration of 2-methoxyestradiol (2ME2), a selective Hif1α inhibitor, attenuated experimental IVD degeneration in mice. The findings of this study show that aberrant Hif1α activation in EP and AF tissues induces pathological changes in DDD, implying that inhibition of aberrant Hif1α activity is a potential therapeutic strategy for DDD.

## Introduction

Low back pain (LBP) is a debilitating condition that affects ~84% of the population in their lifetime.^1^ LBP affects physical function, reduces quality of life, and causes psychological distress.^2^ Although LBP is a multifactorial disease, studies report that intervertebral disc (IVD) degeneration is the main contributing factor. IVD degeneration is a common musculoskeletal disorder caused by multiple factors, such as aging, altered metabolism and genetic predisposition.^3–5^ Current therapies for IVD degeneration mainly include pain relief through physical rehabilitation therapy or surgical treatment. Only a few effective disease-modifying treatments are available for IVD degeneration, as its pathogenic mechanism is not fully known.^6–8^ Understanding the molecular mechanisms of IVD degeneration is important for the development of novel biotherapeutics.

The IVD is a functional unit connecting the vertebral bodies of the spine and is composed of three regions, including a soft gelatinous nucleus pulposus (NP) at the center surrounded by a tough peripheral lamellar annulus fibrosus (AF) and cartilaginous endplates (EPs) located between the NP and the vertebral body.^9^ The IVD is the largest avascular tissue that physiologically lacks blood vessels and the lymphatic system. Cartilaginous EP serves as a selective permeability barrier for metabolic exchanges in the IVD.^10,11^ Under hypoxic conditions, the most characterized cellular response to low oxygen levels is the induction of hypoxia-inducible factors (HIFs) and their resultant responses.^12^ HIFs are heterodimers comprising a constitutively expressed β subunit (HIFβ) and an oxygen-sensitive α-subunit (1α, 2α, and 3α). In the presence of oxygen, the α subunit is hydroxylated on specific proline residues by members of the prolyl-hydroxylase domain (PHD) family. The hydroxylated α subunit is thus recognized and bound by the von Hippel-Lindau (VHL)/E3 ubiquitin ligase and targeted for protein degradation through the proteasome. Under hypoxic conditions, PHD activity is suppressed, and the α subunit escapes proteasomal degradation and forms a heterodimer with the β subunit to regulate the transcription of HIF target genes.^13^

Multiple studies have explored the roles of HIF signaling in IVD homeostasis; however, most studies focus on the functions of HIF1α in NP cells.^14–16^ Studies report that HIF1α is mainly stabilized in the NP, whereas it is absent from EP and AF cells in rats.^17^ Previous studies demonstrated that HIF1α signaling plays an important role in a variety of NP cellular functions in vitro, including cell survival, proliferation, regulation of metabolism, and matrix synthesis.^14,18^ Analysis of conditional knockout mice established using notochord-specific Foxa2^iCre^ indicated that the lack of *Hif1α* in notochordal cells leads to severe cell death and the disappearance of the NP. This finding implies that HIF1α is important for NP formation and survival during IVD development.^19^ Since all IVD components are involved in the pathogenesis of IVD disease through the reciprocal interaction among them, the role of HIF1α in NP development suggests its potential important role in the pathogenesis of NP degeneration and/or IVD. Although an in vitro study suggested a possible association of HIF1α with DDD pathogenesis,^20^ the in vivo functions of HIFs in regulating IVD homeostasis and/or DDD pathogenesis remain elusive. More in vivo studies should be conducted to explore the direct role of HIFs in IVD maintenance and its underlying mechanisms.

In this study, utilizing human degenerative IVD tissues and mouse models of DDD, we found that among the HIF family members, Hif1α expression was significantly upregulated in EP and AF tissues of degenerated discs. To explore the role of Hif1α in DDD pathogenesis, conditional *Vhl* deletion was induced, resulting in aberrant Hif1α activation, in the EP and AF tissues of adult mice. The findings showed that aberrant Hif1α activation caused age-dependent IVD degeneration, partly through increased glycolytic metabolism and suppression of mitochondrial function. Notably, inhibition of Hif1α signaling activity using either a selective Hif1α inhibitor (2ME2) or conditional deletion of *Hif1α* in *Vhl*-deficient mice with DDD alleviated the pathological changes of disc degeneration.

## Results

### Aberrant HIF1α signaling is upregulated in degenerative IVD

**Fig. 1:**
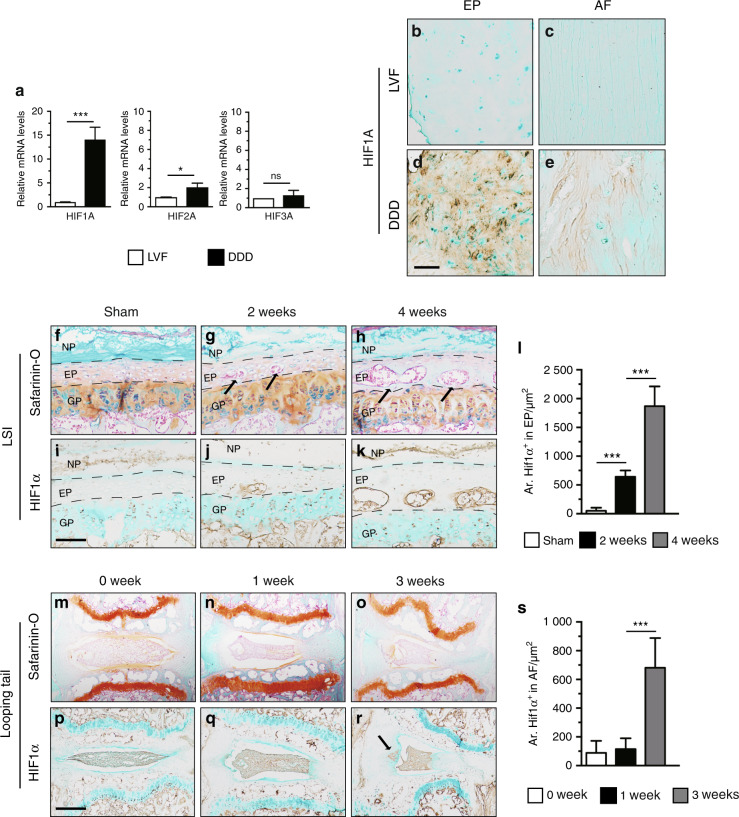
Upregulated HIF1α signaling in EP and AF tissues of degenerative IVD. (**a**) Real-time PCR analysis of *HIF1A, HIF2A* and *HIF3A* expression in degenerative lumbar EP from patients with DDD. **b**–**e** Immunohistochemical detection of HIF1A expression was performed in normal and degenerative lumbar EP and AF tissues. **f**–**h** Histological analysis of IVD samples harvested at 2 and 4 weeks post LSI (lumbar spine instability) surgery and by Safranin O/Fast green staining. The arrowheads indicate EP ossifications. IHC staining for HIF1α was performed in the IVD of wild-type mice. (i) Sham mice, (**j**, **k**) 2 weeks and 4 weeks after LSI surgery. **l** Quantitative analysis of the areas of HIF1α immunoreacted positive cells in LSI surgery. **m**–**o** Caudal vertebrae were harvested at 0, 1, and 3 weeks after tail-looping surgery and analyzed histologically by Safranin O/Fast green staining. **p**–**r** Immunohistochemical analysis of HIF1α^+^ cells in tail-looping surgery. The black arrow indicates AF degeneration. **s** Quantitative analysis of HIF1α^+^ cells. Scale bar: 100 µm (**b**–**e**), 50 µm (**f**–**k**), 100 µm (**m**–**r**). Data are expressed as the percent expression relative to controls. Values represent the mean (symbols) ± SD (error bars). *P < 0.05; **P < 0.01; ***P < 0.001.

To explore the possible relationship between HIF signaling and DDD pathogenesis, the expression levels of HIFs in degenerative EP and AF tissues of humans and two DDD mouse models were determined. Normal EP and AF tissues from patients with lumbar vertebral fracture (LVF) without a documented clinical history of LBP were used as controls. Degenerative lumbar EP and AF tissues from patients with DDD according to the modified Pfirrmann classification system were used as the DDD group (Supplementary Fig 1A–D).^21^ Quantitative real-time (qPCR) analysis of human tissues showed higher expression levels of *HIF1a* in the DDD group than in the control group. Although the fold-increase of *HIF2A* mRNA was statistically significant in the DDD group compared with the control group, the degree of its change was less compared with that of *HIF1A*. The mRNA expression level of *HIF3A* showed no significant difference between the two groups (Fig. 1a). Furthermore, immunohistochemical analysis showed that HIF1A expression was significantly higher in human degenerative EP and AF tissues than in the control tissues (Fig. 1b–e), whereas HIF2A levels were not significantly different between human degenerative EP and AF tissues and the control group (Supplementary Fig 1E).

We next determined the protein levels of HIF1α in two experimental mouse models of DDD. The first model was established using unbalanced mechanical loading in the spine by transecting the L4–L5 spinous processes and the supraspinous and interspinous ligaments in 2-month-old male mice in a process referred to as lumbar spine instability (LSI) surgery (Supplementary Fig. 2A). Safranin O/Fast green staining analysis showed that EPs began to undergo ossification at 2 weeks post surgery, as indicated by the green-stained bone matrix in LSI mice compared with the sham group (Fig. 1f–h). Immunostaining analysis showed that the number of cells positive for HIF1α gradually increased in degenerative EP cartilage at 2 and 4 weeks after LSI surgery compared with that in sham-operated controls (Fig. 1i–k, l). To further explore the role of HIF signaling in DDD, a second IVD degeneration model, referred to as tail-looping surgery, was established by looping the mouse tails in the position between C5 and C13 to mimic aberrant mechanical loading leading to disc degeneration (Supplementary Fig. 2B). Histological analysis of the IVD showed progressive degeneration with the loss of intact structure at 1 and 3 weeks after tail-looping surgery (Fig. 1m–o). In addition, the expression of HIF1a was significantly upregulated in degenerative AF at 3 weeks compared with that at 1 week after tail-looping surgery (Fig. 1p–r, s). In contrast, the expression of HIF2α showed no significant changes after LSI or tail-looping surgery compared with controls (Supplementary Fig. 2C-F). These findings indicate that HIF1α expression in EP and AF tissues is spatiotemporally regulated during DDD pathogenesis, implying a potential essential role of HIF1α signaling in DDD pathogenesis.

### Aberrant HIF1α activation in EP and AF cells through inducible deletion of *Vhl* in Col2a1-positive cells in adult mice

**Fig. 2:**
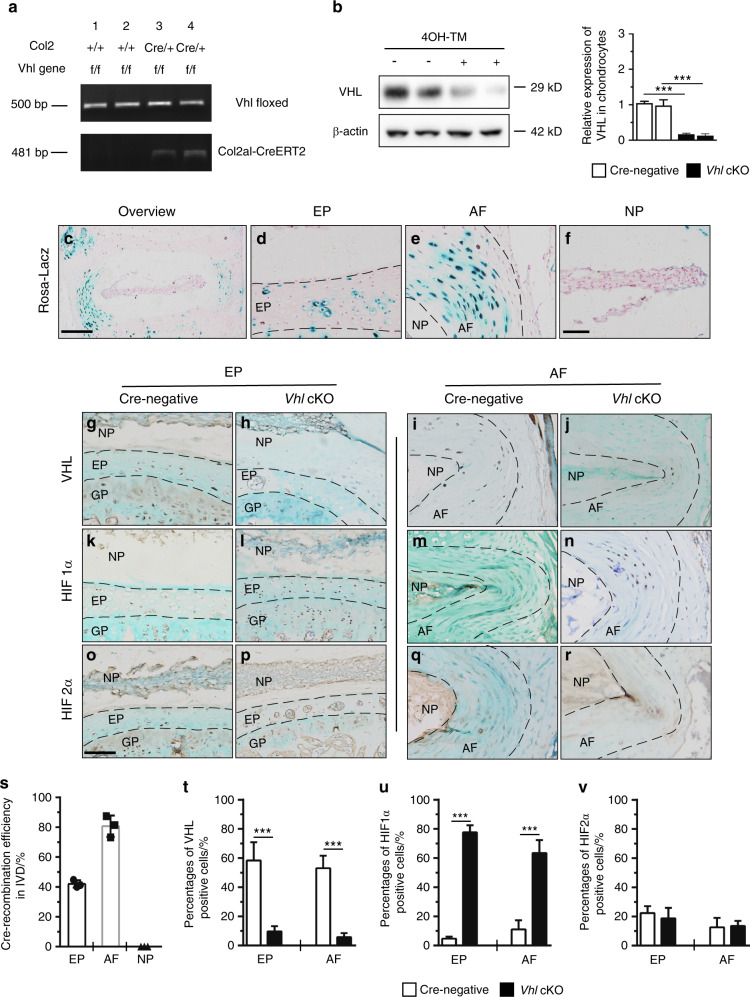
Conditional deletion of Vhl in EP and AF cells leads to aberrant Hif1α activation in mouse IVDs. **a** Polymerase chain reaction (PCR) for genotyping *Vhl*^*flox/flox*^ *Col2a1-CreERT*^*2*^ (*Vhl* cKO) mice. **b** VHL protein expression in response to tamoxifen (TM) treatment in primary IVD cells from *Vhl*^*flox/flox*^ *Col2a1-CreERT*^*2*^ mice, as determined by western blotting. **c**–**f** Lac-Z staining detecting the Cre-mediated recombination efficiency in IVD cells of *Col2a1-CreERT*^*2*^*; Rosa26* mice at 4 months of age (*n* = 5 per group). (**g**–**r**) Immunohistochemistry analysis of VHL, HIF1α and HIF2α in the EP and AF of *Vhl* cKO and control mice (*n* = 5 per group). **s** Quantitative analysis of the Cre recombination efficiency in IVD cells. **t**–**v** Quantitative analysis of VHL^+^ cells and HIF1α^+^ and HIF2α^+^ cells in the EP and AF of *Vhl* cKO and control mice. Scale bar: 100 µm (**c**), 50 µm (**d**–**r**). Data are expressed as the percent expression relative to controls. Values represent the mean (symbols) ± SD (error bars). *P *<* 0.05; **P *<* 0.01; ***P *<* 0.001*.*

To further explore the role of HIF signaling in IVD maintenance in vivo, the HIF pathway was hyperactivated by conditionally deleting the HIF-negative regulator VHL in chondrocytes (*Vhl*^*flox/flox*^ *Col2a1-CreERT*^*2*^ mice; hereafter, *Vhl* cKO mice) (Fig. 2a, b). *Col2a1-CreERT*^*2*^ transgenic mice were crossed with *Rosa26* reporter mice to determine the Cre-mediated recombination efficiency in IVD tissues. Tamoxifen (TM) was administered to mice at the age of 2 months, and Lac-Z staining of tissues was performed when mice were 4 months old. Cre recombination efficiency in AF cells (80.6%) and EP cartilage cells (42.1%) was observed, whereas few Lac-Z-positive cells were located in the nucleus pulposus cells, indicating that the *Vhl* gene in AF cells and EP chondrocytes at the adult stage had been successfully deleted (Fig. 2c–f, s). IHC analysis was performed to determine the expression levels of VHL-HIF signaling-related molecules in 4-month-old *Vhl* cKO mice. Immunohistochemical staining confirmed the loss of VHL expression in most AF cells and EP cells in conditional knockout mice compared with Cre-negative mice (Fig. 2g–j, t). Deletion of *Vhl* caused significant upregulation of HIF1α expression in EP and AF cells of *Vhl* cKO mice compared with Cre-negative mice (Fig. 2k–n, u). Notably, immunostaining analysis showed that the expression level of HIF2α was not significantly different between *Vhl* cKO and Cre-negative mice (Fig. 2o–r, v).

### Aberrant Hif1α activation causes age-dependent IVD degeneration

**Fig. 3:**
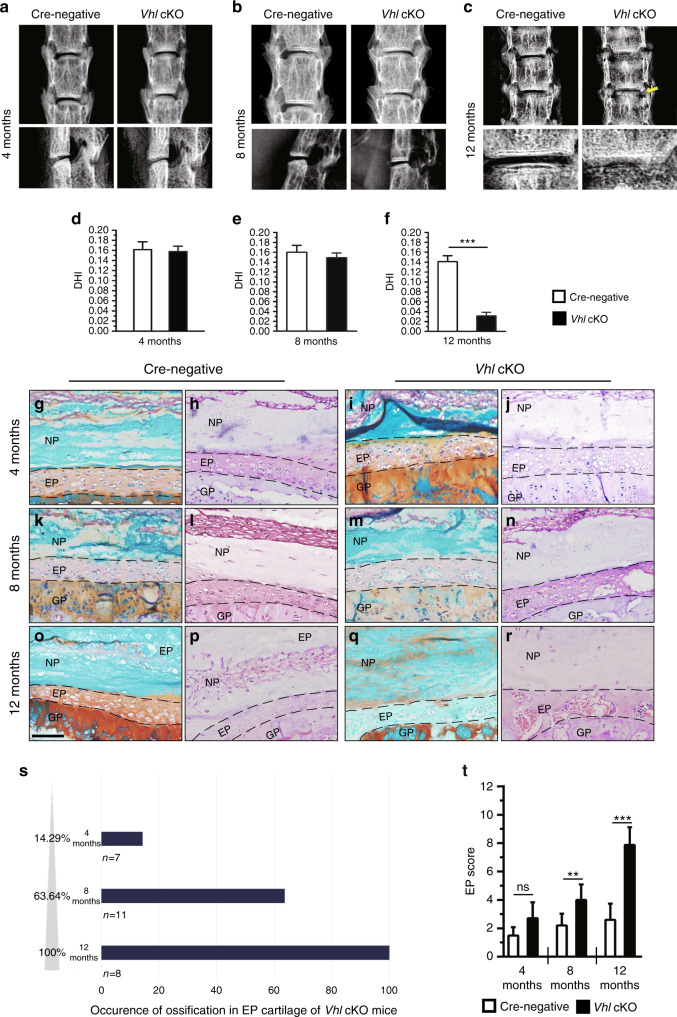
Aberrant Hif1α activation in IVD cells causes age-dependent degeneration. **a**–**c** Radiographic assessment of IVD phenotypes in *Vhl* cKO mice and control mice at 4, 8, and 12 months of age. The yellow arrow indicates the formation of osteophytes (*n* = 7–8 per group). **d**–**f** Methods for measurement of the lengths between L4 and L5 (L4/L5) and for calculation of the disc height index (DHI). **g**–**r** Safranin-O/Fast green/H&E staining of lumbar IVDs at 4, 8, and 12 months (*n* = 7–8). **s** Occurrence of ossification in EP cartilage of *Vhl cKO* mice. **t** Histological degenerative scores of EP cartilage in WT and *Vhl* cKO mice. Scale bar: 50 µm (**g**–**r**). Data are expressed as the percent expression relative to controls. Values represent the mean (symbols) ± SD (error bars). *P < 0.05; **P < 0.01; ***P < 0.001.

**Fig. 4:**
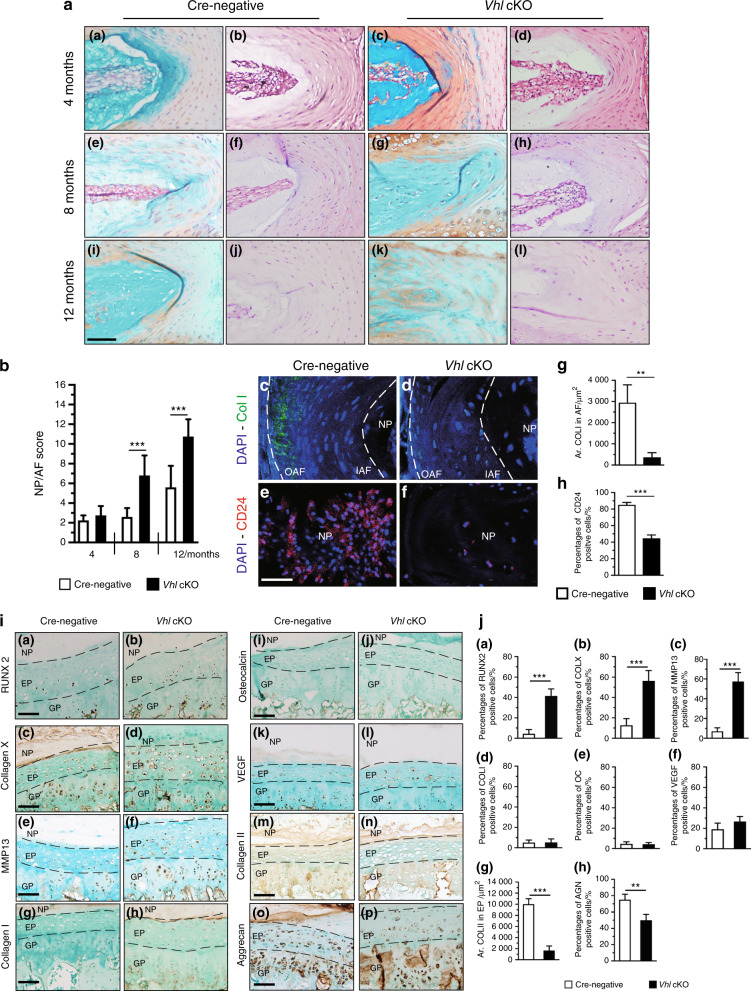
Aberrant Hif1α activation causes degenerative changes in IVD tissues. **a** Fast green/Safranin O- and H&E-stained coronal sections of the AF and NP from WT and *Vhl* cKO mice. **b** Histological degenerative scores of NP/AF tissues in WT and *Vhl* cKO mice. **c**, **d** Representative COLI immunofluorescence images of IVDs from the lumbar AF of 12-month-old WT and *Vhl* cKO mice (green: anti-ColI; blue: Hoechst) (*n* = 3–4). **e**, **f** CD24 signals were analyzed by immunofluorescence assay from NP cells of WT and *Vhl* cKO mice at the age of 12 months. (red: anti-CD24; blue: Hoechst) (*n* = 3–4). **g**–**h** Quantitative analysis of COLI^+^ cells and CD24^+^ cells in the EP and AF of *Vhl* cKO and control mice. **i** Immunostaining and **j** quantitative analysis of RUNX2^+^ cells, COLX^+^ cells, MMP13^+^ cells, COLI^+^ cells, OC^+^ cells, VEGF^+^ cells, COL2AL and aggrecan in lumbar discs from WT and Vhl cKO mice at the age of 4 months (*n* = 3 per group). Scale bar: 50 µm (**a**, **c**–**f**, **i**). Data are expressed as the percent expression relative to controls. Values represent the mean (symbols) ± SD (error bars). *P *<* 0.05; **P *<* 0.01; ***P *<* 0.001*.*

Two-month-old *Vhl* cKO and Cre-negative mice were injected with TM for 5 days. IVD samples were harvested at 4, 8 and 12 months of age. Previous studies have reported that mice with *Vhl* loss in chondrocytes exhibit a severe dwarfism phenotype.^22^ In the current study, mice with chondrocyte-specific deletion of *Vhl* at the adult stage showed no significant gross change 2 months after TM injection (data not shown). The disc height index (DHI) between L4 and L5 was measured in *Vhl* cKO and control mice under X-ray view of the lumbar spine following a method described previously.^23^ Analysis showed no significant difference in the DHI between the two groups at 4 and 8 months (Fig. 3a, b, d, e), whereas the DHI was significantly lower in *Vhl* cKO mice at 12 months than in control mice (Fig. 3c, f). Furthermore, osteophyte formation was observed in the lumbar spine of *Vhl* cKO mice at 12 months (Fig. 3c). Discs of *Vhl* cKO and Cre-negative mice were explored using hematoxylin & eosin (H&E) and Safranin O/Fast green staining at multiple stages. At 4 months, histological analysis showed early signs of degeneration of the lumbar IVDs in *Vhl* cKO mice, with a higher number of enlarged chondrocytes in the EP cartilage, indicating the presence of chondrocyte hypertrophy in EP cartilage (Fig. 3g–j). In addition, higher expression of type X collagen (COLX) and matrix metalloproteinase 13 (MMP 13) in EP cartilage in *Vhl* cKO mice implied the development of chondrocyte hypertrophy (Fig. 4I c–f). At 8 months, histological analysis showed that EP cartilage began to undergo ossification (Fig. 3k–n). At 12 months, analysis showed severe degeneration of lumbar IVDs in *Vhl* cKO mice compared with the control group. Moreover, EP cartilage was almost completely replaced by bone tissue in IVDs of *Vhl* cKO mice (Fig. 3o–r). Notably, the occurrence of ossification in the EP cartilage of *Vhl* cKO mice was age-dependent (Fig. 3s). Furthermore, the EP degenerative histological score of *Vhl* cKO mice was higher than that of Cre-negative mice at 8 and 12 months (Fig. 3t).

Furthermore, the role of aberrant Hif1α activation on the AF and NP degeneration that occurs during the aging process was explored by analysis of *Vhl cKO* mice using H&E and Safranin O/Fast green staining at multiple stages. Histological analysis of NP and AF showed no significant difference between *Vhl* cKO and Cre-negative mice after 4 months [Fig. 4a, panels (a)–(d), 4b] At 8 and 12 months, the NP and AF of *Vhl* cKO mice showed more Safranin O-positive staining with the appearance of some chondrocyte-like cells, indicating the presence of degenerative changes. H&E staining showed disorganized AF structure, loss of the NP-AF boundary, and signs of fibrosis in the NP and AF of *Vhl* cKO mice [Fig. 4a, panels (e)–(l)]. The NP-AF degenerative histological score of *Vhl* cKO mice was higher than that of Cre-negative mice at 8 and 12 months (Fig. 4b). Furthermore, immunofluorescence (IF) analysis of NP and AF in 12-month-old *Vhl* cKO mice and Cre-negative mice was performed. Under physiological conditions, type I collagen (ColI) is mainly expressed in the outer AF. In this study, analysis showed the expression of ColI in the outer AF of 12-month-old Cre-negative mice; however, ColI was not expressed in the AF of *Vhl* cKO mice (Fig. 4c, d, g). CD24 is a marker for NP cells; control NP cells tested positive for CD24, whereas *Vhl* cKO mice showed few CD24 signals in NP cells (Fig. 4e, f, h). These findings indicate that aberrant Hif1α activation causes age-dependent IVD degeneration.

Chondrocyte hypertrophy plays an important role in endochondral ossification during skeletal development.^24^ Notably, studies report that chondrocyte hypertrophy induces the initiation and progression of EP cartilage degeneration during DDD development.^25^ To explore the mechanism of the accelerated DDD in VHL-conditional-knockout mice, the expression levels of proteins related to chondrocyte hypertrophy were explored by IHC staining. COLX and MMP13, catabolic enzymes for cartilage extracellular matrix (ECM), are important markers of hypertrophic chondrocytes.^26^ The transcription factor RUNX2 modulates chondrocyte hypertrophy and the expression of ECM-degrading enzymes.^27^ Analysis showed a significant increase in the expression levels of COLX, MMP13, and RUNX2 in the EP cartilage of Vhl cKO mice at the age of 4 months compared with controls [Fig. 4i, panels (a)–(f), Fig. 4j, panels (a)–(c)]. In contrast, the levels of vascular endothelial growth factor (VEGF), COLI and osteocalcin (OC), which are normally detected in ossified bone, were negligible [Fig. 4i, panels (g)–(l), 4j, panels (d)–(f)].^28,29^ Type II collagen (ColII) and aggrecan expression levels were significantly lower in mutant mice than in control mice at the age of 4 months [Fig. 4i, panels (m)–(p), 4j, panels (g)–(h)]. In addition, IHC staining was performed to explore pathological changes in EP cartilage at the age of 8 months. IHC analysis showed higher expression levels of COLX, MMP13, RUNX2, VEGF, COLI, and OC in the EP cartilage of Vhl cKO mice compared with the levels in Cre-negative mice (Supplementary Fig 3A, B). These findings indicate that *Vhl* deficiency promotes hypertrophy and ossification in EP, which may directly disrupt EP cartilage homeostasis during DDD development.

### Aberrant HIF1α signaling enhances glycolytic metabolism and suppresses mitochondrial activity in DDD pathogenesis

**Fig. 5:**
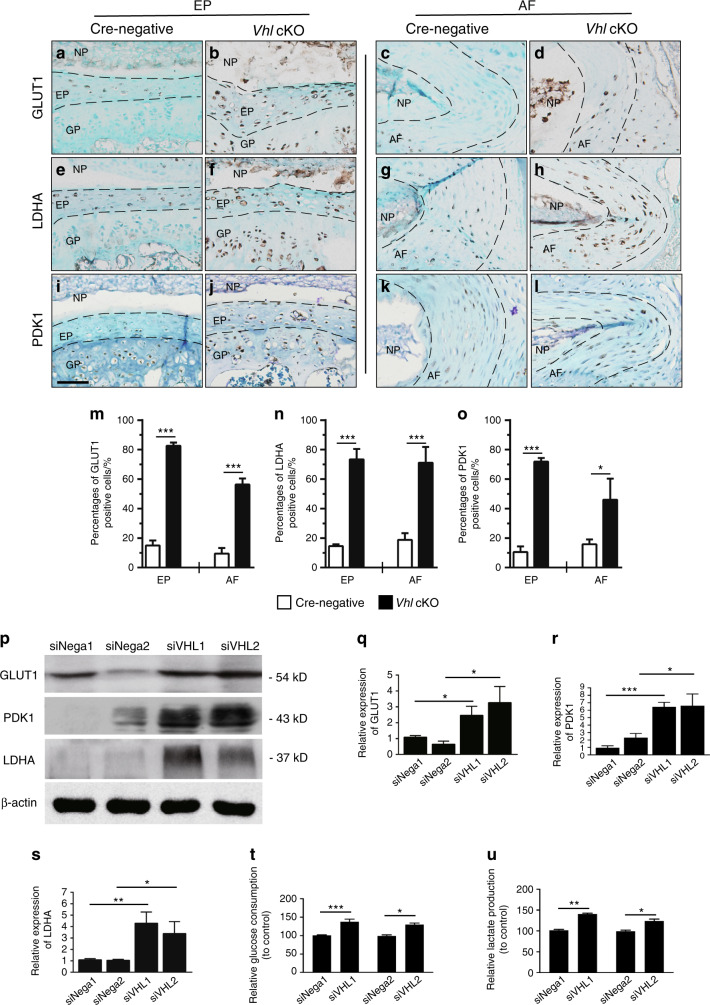
Aberrant HIF1α signaling enhances glycolytic metabolism. **a**–**l** Immunohistochemical detection of GLUT1, LDHA, and PDK1 expression in lumbar discs of WT and Vhl cKO mice (*n* = 3–5 per group). **m**–**o** Percentages of GLUT1-, LDHA- and PDK1-immunoreacted positive cells. **m**, GLUT1-; **n**, LDHA-; and **o**, PDK1-positive cells were quantified. **p** Western blotting analysis of GLUT1, PDK1, and LDHA expression in iAF cells in the siRNA-Vhl group, where the siNega group was used as a control. **q**–**s** The signal intensities were quantified (*n* = 3 per group). **t**–**u** The glucose consumption and lactate production of iAF cells transfected with siRNA-Vhl were determined. Scale bar: 50 µm (**a**–**l**). Data are expressed as the percent expression relative to controls. Values represent the mean (symbols) ± SD (error bars). *P *<* 0.05; **P *<* 0.01; ***P *<* 0.001*.*

Cell metabolism is the basis of cell survival and function. Cells can respond to environmental stress, such as hypoxia, by downregulating energy-demanding processes.^30^ Previous studies have shown that glycolysis is the main source of energy in IVD cells.^31^ Energy production in degenerated discs is different from that in normal discs and is associated with high lactic acid levels resulting from anaerobic glycolysis metabolism.^32^ These findings indicate that glycolytic metabolism plays an important role in maintaining homeostasis of the IVD. Hif1α is implicated in the regulation of glycolytic metabolism in bone tissue by direct regulation of multiple key enzymes involved in the glycolytic process.^33^ To explore the potential effects of aberrant HIF1α activation on glycolytic metabolism in the IVD of *Vhl* cKO mice, the expression level and distribution of glucose transporter 1 (GLUT1), an HIF1α target gene, was determined. A previous study reported that only NP cells express moderate GLUT1 protein, whereas AF and EP cells have low GLUT1 levels.^20^ Analysis showed that aberrant HIF1α signaling significantly increased GLUT1 expression in EP and AF tissues in *Vhl* cKO mice compared with control mice (Fig. 5a–d, m). Furthermore, the expression levels of two glycolysis-related enzymes, namely, pyruvate dehydrogenase kinase I (PDK1) and lactate dehydrogenase A (LDHA), were explored. Analysis showed significantly higher expression levels of PDK1 and LDHA in EP and AF tissues in *Vhl* cKO mice than in Cre-negative mice (Fig. 5e–l, n, o). The iAF cell line, which is a cell model used for studying AF cells, was used for in vitro analysis.^34^ iAF cells were transfected with siRNA-Vhl (iAF cells transfected with siRNA-Vhl are referred to as iAF-siRNA-Vhl, and cells transfected only with the vehicle are referred to as iAF-siNega) to induce activation of HIF1α (Supplementary Fig 3D). Consistently, western blotting analysis showed upregulation of GLUT1, PDK1, and LDHA following VHL knockdown in iAF cells (Fig. 5p–s). In addition, a higher rate of glucose consumption and lactate production was observed in iAF-siRNA-Vhl cells than in iAF-siNega cells (Fig. 5t–u). These findings indicate that aberrant Hif1α activation increases glycolytic metabolism in IVDs in vivo and in vitro.

**Fig. 6:**
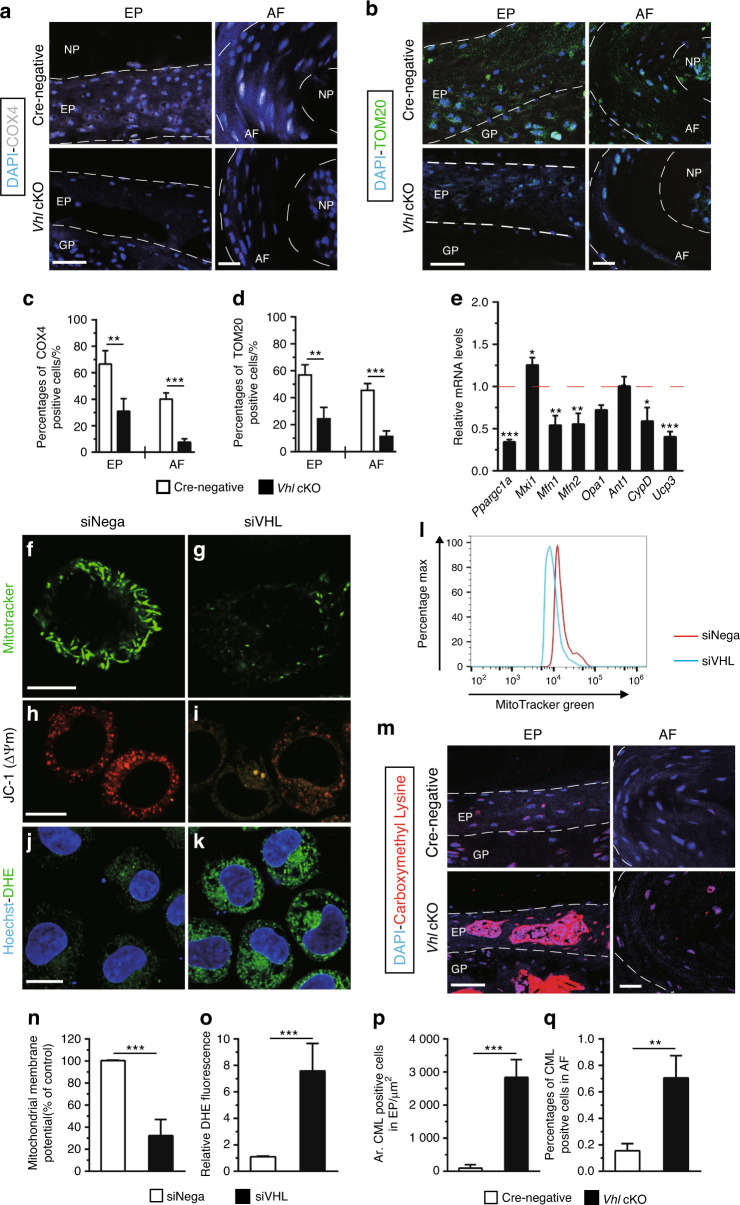
Aberrant HIF1α signaling causes mitochondrial dysfunction. **a**, **b** Immunofluorescence detection of COX4 and TOM20 was performed in lumbar discs of WT and *Vhl* cKO mice (*n* = 3 per group). **c**, **d** Percentages of COX4- and TOM20-immunoreacted positive cells. **e** RCS cells were transfected with siRNA-Vhl (RCS-siRNA-Vhl). Total RNA was isolated, and the mRNA levels of *Ppargc1a*, *Mxi1*, *Mfn1*, *Mfn2*, *Opa1*, *Ant1*, *CypD*, and *Ucp3* were detected by RT-PCR (*n* = 3 per group). **f**, **g** Fluorescent staining for monitoring mitochondria with a MitoTracker® probe. (h-i) RCS cells subjected to siRNA-Vhl were stained with JC-1. Changes in △Ψm were detected by fluorescence microscopy. (**j**, **k**) ROS production was determined using the fluorescent probe dihydroethidium (DHE) (green: DHE; blue: Hoechst) (*n* = 3–4). **l** Quantification of flow cytometry for mitochondrial mass. **m** Carboxymethyl lysine (CML) signals were analyzed by an immunofluorescence assay from WT and *Vhl* cKO mice at the age of 12 months (red: anti-CML; blue: Hoechst) (*n* = 3–4). n Quantification of mitochondrial membrane potential, **o** DHE fluorescence, **p** areas of CML-positive cells in EP, and **q** percentages of CML-positive cells in AF. Scale bar: 50 µm (**a**–**b**, **m**), 20 µm (**h**–**k**), 10 µm (**f**, **g**). Data are expressed as the percent expression relative to controls. Values represent the mean (symbols) ± SD (error bars). *P *<* 0.05; **P *<* 0.01; ***P *<* 0.001*.*

Previous studies report that aberrant Hif1α activation promotes glycolytic processes, which can induce mitochondrial dysfunction.^35,36^ Mitochondrial dysfunction is highly correlated with IVD degeneration.^37,38^ To further explore the mechanistic basis for IVD degeneration resulting from aberrant Hif1α activation, the protein levels of complex IV (cytochrome c oxidase IV, COX4) and TOM20, which are widely used markers for assessing mitochondrial activity, were determined.^36^ IF staining showed significantly lower COX4 and TOM20 expression levels in *Vhl* cKO mice than in Cre-negative mice at the age of 8 months (Fig. 6a–d). To elucidate the mechanisms underlying mitochondrial abnormalities, the expression of genes involved in mitochondrial homeostasis and function in a cultured rat chondrosarcoma (RCS) cell line was explored. The RCS cell line maintains cartilage-like characteristics in vitro and is commonly used for studying EP cartilage cells in vitro.^39^ RCS cells were transfected with siRNA-Vhl (RCS-siRNA-Vhl) to induce HIF1α activation (Supplementary Fig. 3C). RT-PCR results of RCS-siRNA-Vhl showed that mitochondrial biogenesis genes, including peroxisome proliferative activated receptor, coactivator 1a (*Ppargc1a*) and increased expression of max interacting protein 1 (*Mxi1*), were downregulated. These genes are implicated in negatively regulating mitochondrial biogenesis.^40^ Furthermore, knockdown of the *Vhl* gene in RCS cells decreased the mRNA levels of the mitochondrial fusion genes mitofusin-1 (*Mfn1*), mitofusin-2 (*Mfn2*), and optic atrophy 1 (*Opa1*).^41^ The RCS-siRNA-Vhl group showed low expression levels of cyclophilin D (*Ppif* or *CypD*) without significant changes in mitochondrial adenine nucleotide translocator (*Ant1*), which is associated with the mitochondrial permeability transition pore (mPTP).^42^ Moreover, the RCS-siRNA-Vhl group showed significant downregulation of anion carrier uncoupling protein 3 (*Ucp3*), which is involved in the suppression of reactive oxygen species (ROS) levels (Fig. 6e).^43^ Monitoring of mitochondria with MitoTracker Green showed the collapsed morphology of mitochondria in the RCS-siRNA-Vhl group compared with the control group (Fig. 6f–g). Flow cytometry analysis showed that knockdown of *Vhl* decreased mitochondrial mass compared with that of the control group (Fig. 6l). Furthermore, a JC-1 assay was performed to assess the mitochondrial membrane potential (△Ψm). In the RCS-siNega group, JC-1 aggregated in normal mitochondria as red fluorescence. Knockdown of the *vhl* gene caused dissipation of ΔΨm, which was shown by increased green fluorescence (Fig. 6h–i, n). Dissipation of △Ψm is associated with mitochondrial ROS production, and multiple studies report that oxidative stress plays an important role in IVD degeneration.^38^ ROS production levels were determined using the fluorescent probe dihydroethidium (DHE). Analysis showed that the RCS-siRNA-Vhl group had significantly higher ROS levels than the RCS-siNega group (Fig. 6j, k, o). Furthermore, IF staining showed significantly higher expression levels of carboxymethyl lysine (CML) in EP and AF tissue of *Vhl* cKO mice compared with the levels in control mice at the age of 8 months, indicating high levels of oxidative protein damage (Fig. 6m, p, q).^44^ These findings show that aberrant Hif1α activation in *Vhl* cKO mice induced mitochondrial dysfunction during DDD progression.

**Fig. 7:**
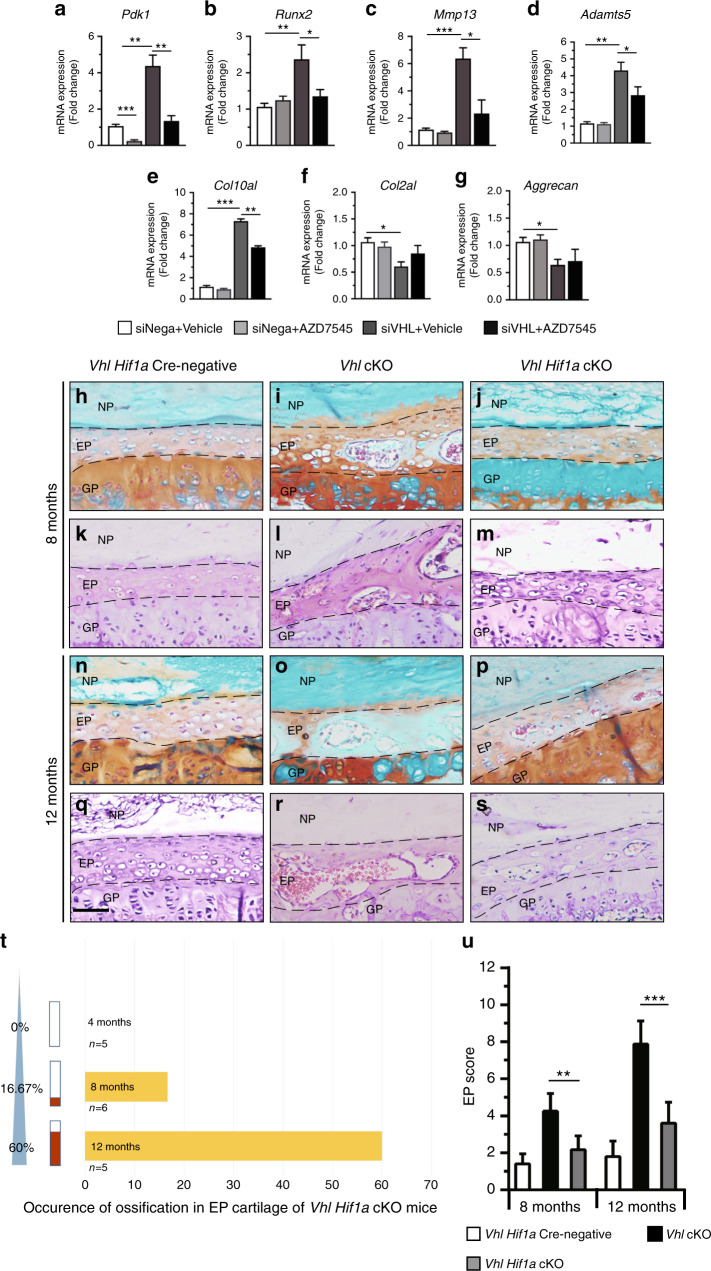
Genetic ablation of the Hif1α gene delays DDD pathogenesis. **a**–**g** RCS cells were transfected with siRNA-Vhl (RCS-siRNA-Vhl), followed by treatment with ADZ7545 (10 μmol·L^−1^). Total RNA was isolated, and the mRNA levels of *Pdk1*, *Runx2*, *Mmp13*, *Adamts5, Col10al*, *Col2al,* and *Aggrecan* were detected by RT-PCR (*n* = 3 per group). **h**–**s** Representative Fast green/Safranin O- and H&E-stained images of EPs from WT, *Vhl* cKO, and *Vhl Hif1α* cKO mice (*n* = 6–8 per group). **t** Occurrence of ossification in EP cartilage of *Vhl Hif1α cKO* mice. **u** Degenerative scores of EP cartilage in WT, *Vhl* cKO, and *Vhl Hif1α cKO* mice. Scale bar: 50 µm (**a**–**l**). Data are expressed as the percent expression relative to controls. Values represent the mean (symbols) ± SD (error bars). *P < 0.05; **P < 0.01; ***P < 0.001.

The enhanced glycolysis program is a potential mechanism underlying the aggravated DDD development observed in VHL mutant mice in which the matrix is broken down by proteinases such as MMP13 and ADAMTS5 produced by degenerative disc cells, with decreased levels of collagen II and proteoglycan.^45^ Then, we investigated whether DDD progression could be alleviated by inhibiting glycolysis. AZD7545 is an inhibitor of pyruvate dehydrogenase kinase,^46^ as evidenced by the reduction in PDK1 expression in the RCS cell line treated with it (Fig. 7a). Although PDK1 signaling was effectively inhibited, the analysis showed no significant changes in *Mmp13*, *Adamts5*, *Runx2*, *Col10a1*, *Col2a1,* and *Aggrecan* levels in the RCS-siNega group after treatment with the PDK1 inhibitor (Fig. 7b–g). The expression levels of *Mmp13*, *Adamts5*, *Runx2* and *Col10a1* were upregulated, whereas the expression levels of *Col2a1* and Aggrecan were downregulated in RCS-siRNA-Vhl cell lines compared with the control (Fig. 7b–g). This finding indicates that chondrocyte hypertrophy and catabolism are induced by aberrant Hif1α activation, which plays important roles in DDD development.^25^ Treatment with AZD7545 alleviated the increased expression levels of *Runx2*, *Mmp13*, *Adamts5*, and *Col10a1* in Vhl-deficient RCS cells (Fig. 7b–e). However, no significant changes were observed in *Col2a1* or aggrecan levels in Vhl-deficient RCS cells treated with the glycolysis inhibitor compared with those treated with the vehicle (Fig. 7f, g). These findings imply that aberrant Hif1α signaling induces chondrocyte hypertrophy and catabolism partly through activation of glycolytic metabolism.

### Genetic ablation of the *Hif1α* gene alleviates DDD pathogenesis in *Vhl*-deficient mice

To further explore the functional role of aberrant Hif1α activation in DDD progression in *Vhl* cKO mice, *Col2al-CreERT2*; *Vhl*^*flox/flox*^ mice were crossed with *Hif1α*^*flox/flox*^ mice to establish *Col2al-CreER*^*T2*^; *Vhl*^*flox/flox*^; *Hif1α*^*flox/flox*^ double KO mice (Supplementary Fig. 4A, B). WB analysis showed that the protein levels of VHL and HIF1α following TM treatment were significantly lower in primary IVD cells from *Vhl*^*flox/flox*^; *Hif1α*^*flox/flox*^ *Col2al-CreER*^*T2*^ mice than in those from control mice (Supplementary Fig. 4C). IHC staining showed effective loss of VHL and HIF1α levels in AF cells and EP cells of double knockout mice at the age of 4 months, whereas the levels in control mice were not affected (Supplementary Fig. 4D–F). Notably, the expression levels of HIF2α were not significantly different between double knockout mice and control mice (Supplementary Fig. 5A, D). X-ray studies showed that deletion of *Hif1α* in *Vhl* cKO mice partially rescued the narrowing of spaces between vertebrae of *Vhl* cKO mice at 12 months (Supplementary Fig. 5B, C, E, F). Histological analysis showed that deletion of the *Hif1α* gene in *Vhl* cKO mice significantly ameliorated EP degeneration at 8 months and 12 months (Fig. 7h–s). Moreover, deletion of the *Hif1α* gene in *Vhl*-deficient mice partially decreased the expression levels of OC, RUNX2, COLI, and VEGF (Supplementary Fig. 6A–P). Double KO mice showed a lower occurrence of ossification in EP tissue than *Vhl* cKO mice (Fig. 7t). In addition, the quantitative score of EP degeneration in double mutant mice was significantly lower than that of *Vhl* mutants at 8 and 12 months; however, it was higher than that of Cre-negative mice (Fig. 7u). Analysis of NP-AF degeneration showed that the disorganized structure of NP and AF tissues observed in *Vhl* cKO mice was partially attenuated in 8-month-old and 12-month-old double KO mice (Supplementary Fig. 7A-L). Quantitative analysis with the NP-AF histological scoring system showed similar results (Supplementary Fig. 7M). IF staining showed significant upregulation of CD24 and ColI expression after deletion of the *Hif1α* gene in 12-month-old *Vhl/Hif1α* cKO mice (Supplementary Fig. 7N–S, G–J).

Furthermore, changes in glycolysis-related genes in *Vhl*/*Hif1α* double KO mice at the age of 8 months were explored. Analysis showed that double mutant discs had low expression levels of glycolytic genes (Supplementary Fig. 8A, C, D). Furthermore, the mitochondrial status in *Vhl*/*Hif1α* mutants was explored. IF staining analysis showed that deletion of the *Hif1α* gene in *Vhl* cKO mice restored mitochondrial activity, which was indicated by the higher expression levels of COX4 and TOM20 and lower expression levels of CML in EP and AF tissues compared with the levels in control mice (Supplementary Fig 8B, E–H). These findings indicate that deletion of the *Hif1α* gene partially rescued IVD degeneration in *Vhl* cKO mice.

### Pharmacologic inhibition of Hif1α attenuates experimental IVD degeneration in mice

**Fig. 8:**
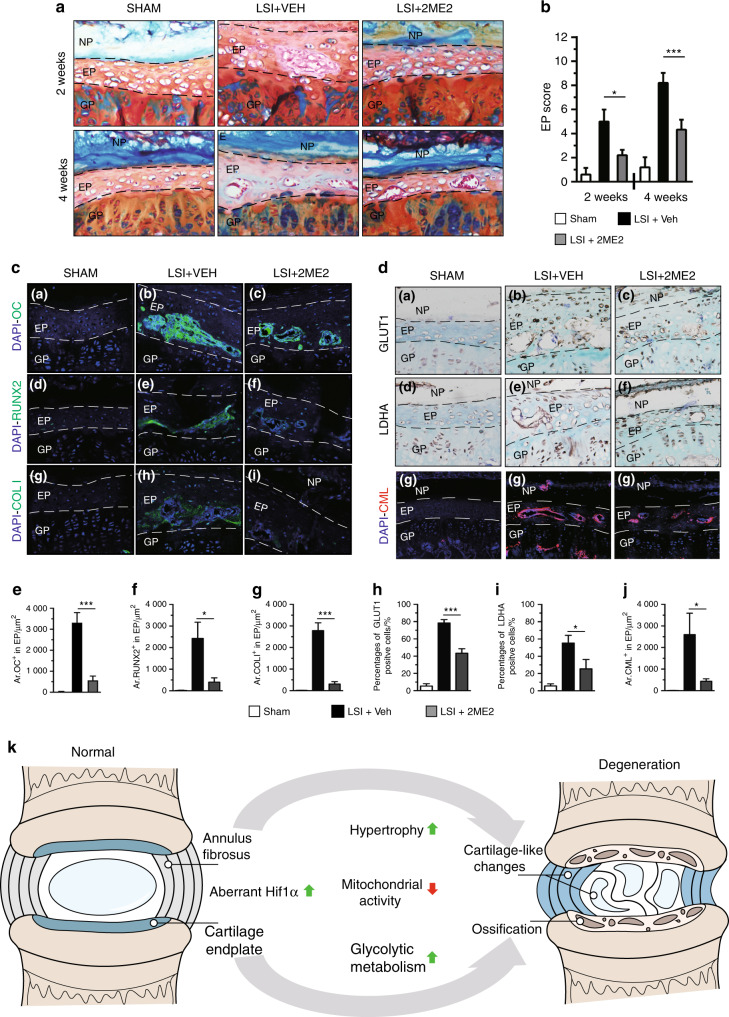
Pharmacologic inhibition of Hif1α attenuates experimental IVD degeneration. **a** IVD samples were harvested at 2 and 4 weeks post LSI surgery and analyzed histologically by Safranin O/Fast green/alcian blue staining. **b** Histological degenerative scores of EP tissue. **c** Immunofluorescence detection and **e**–**g** quantitative analysis of OC, RUNX2, and COLI expression were performed in lumbar discs of mice at 4 weeks post LSI surgery (*n* = 3 per group). **d** Immunostaining and **h**–**j** quantitative analysis of GLUT^+^ cells, LDHA^+^ cells and CML^+^ cells (red) in lumbar discs (*n* = 3 per group). **k** Schematic diagram of the mechanisms underlying the roles of aberrant HIF1α signaling in DDD pathogenesis. Scale bar: 50 µm (**a**, **c**, **d**). Data are expressed as the percent expression relative to controls. Values represent the mean (symbols) ± SD (error bars). *P *<* 0.05; **P *<* 0.01; ***P *<* 0.001*.*

Further analysis was performed to explore whether aberrant Hif1α activation is a potential therapeutic target for DDD. The effect of 2-methoxyestradiol (2ME2), an Hif1α inhibitor, on DDD pathogenesis in C57BL/6J mice was explored.^47^ Different groups of mice were given multiple doses of 2ME2 to determine the optimal dose, which was identified as 75 mg per kg body weight (Supplementary Fig 9A-F). X-ray analysis showed that the DHI between L4 and L5 was significantly higher in 2ME2-treated LSI mice than in vehicle-treated LSI mice 4 weeks after surgery (Supplementary Fig 9G, H). Furthermore, histological analysis was performed on the discs of sham, LSI-operated mice treated with either vehicle or 2ME2 using H&E staining and Safranin O/Fast green staining. Analysis showed that 2ME2 partially alleviated LSI-induced ossification of EP tissues at 2 weeks and 4 weeks after surgery (Fig. 8a) (Supplementary Fig 10A–F). Consistent with the histological findings, EP degenerative scoring analysis showed that the Hif1α inhibitor significantly alleviated IVD degeneration in LSI-operated mice (Fig. 8b). Furthermore, low Hif1α expression levels were observed in EP cells of 2ME2-treated LSI mice (Supplementary Fig 10G–I, P), whereas HIF2α expression levels in 2ME2-treated LSI mice were similar to those of controls, as shown by IHC staining (Supplementary Fig 10J–L, Q). Consistently, the expression levels of OC, RUNX2 and COLI were significantly decreased in the 2ME2-treated group [Fig. 8c, panels (a)–(i), 8e–g]. Moreover, LSI mice treated with 2ME2 showed a significant reduction in the expression levels of glycolytic metabolism-related genes [Fig. 8d, panels (a)–(f), 8h, i]. Furthermore, analysis of the mitochondrial status showed that the COX4 expression level was upregulated, whereas the CML expression level was downregulated in EP cells of 2ME2-treated LSI mice compared with the levels in vehicle-treated LSI mice [Fig. 8d, panels (g)–(i), 8j] (Supplementary Fig 10M–O, R). These findings indicate that Hif1α is a potential therapeutic target for DDD (Fig. 8k).

## Discussion

LBP is a common disease associated with aging and abnormal mechanical loading. IVD degeneration is a major contributing factor for LBP and is characterized by disc height decrease, disorganized structure of NP and AF tissues, and EP ossification.^48^ To prevent IVD degeneration and facilitate the development of disease-modifying therapies for IVD, it is important to explore the molecular and cellular mechanisms of IVD homeostasis. The IVD is one of the largest avascular organs in humans in which HIFs mainly govern the expression of several genes that mediate cellular adaptation.^9^ The mechanism of HIFs in modulating IVD homeostasis has not been fully clarified, and most studies mainly focus on the physiological role of Hif1α in NP tissue in vitro.^14–20^ HIFα has three isoforms, namely, HIF1α, HIF2α, and HIF3α, and each is implicated in the modulation of different physiological functions in a wide range of cells/organs. HIF1α and HIF2α exhibit antagonist effects on OA pathogenesis. Loss of *Hif1α* in chondrocytes accelerates OA progression and induces increased MMP13 expression in mice.^49^ Transgenic mice overexpressing HIF2α show spontaneous cartilage destruction.^50^ Our group also found that upregulated Hif2α led to accelerated development of age-associated and surgically induced OA in *Vhl*-deficient mice.^51^ However, only a few studies have explored the in vivo role of HIFα in regulating DDD pathogenesis at the adult stage.^19^ The findings of this study showed aberrant HIF1α signaling in EP and AF tissues of degenerated human and mouse discs. Although HIF2α mRNA levels were significantly higher in the DDD group of human samples than in the control group, no significant changes in HIF2α protein levels were observed in degenerative EP and AF tissues of *Vhl* cKO mice. Mechanical loading is an important risk factor for DDD. In addition, mechanical stress induces Hif1α expression in cartilage.^52^ The findings of the current study showed that pathological mechanical loading induced by the tail-looping model and LSI model on the spine caused aberrant HIF1α signaling in EP and AF tissues, resulting in disc degeneration. *Hif1α* activity was genetically hyperactivated by generating a mouse model with conditional deletion of *Vhl* in EP and AF tissues. This model displayed a phenotype with similar features to those of human disc degeneration, such as chondrocyte hypertrophy, severe loss of proteoglycan in EP cartilage, disorganized NP/AF structure, and osteophyte formation. Although NP cells were not directly targeted by *Col2a1-CreER*^*T2*^, *Vhl* cKO mice exhibited NP degeneration features. This finding implies that the NP degeneration observed in *Vhl* cKO mice was secondary to EP ossification, which may have disrupted the nutrient supply to NP tissues and may have inhibited the removal of metabolic waste from NP tissue. These findings indicate that aberrant HIF1α signaling in EP and AF cells may cause IVD degeneration.

The VHL-HIF signaling pathway has multiple downstream target molecules implicated in the modulation of angiogenesis and cellular metabolism.^53^ Most mammalian cells meet their energetic requirements mainly through mitochondrial oxidative phosphorylation in the presence of oxygen and rely on glycolysis in the cytosol when oxygen levels are low or when HIF proteins are upregulated.^54^ Under normal physiological conditions, stabilized HIF1α drives glycolytic metabolism regularly in NP cells and low levels of glycolytic metabolism in outer AF and EP cells of cultured IVDs.^17^ However, under pathological conditions, as we found in the present study, activated HIF1α signaling led to an aberrant glycolytic program in degenerative discs. We further investigated whether catabolic events in DDD could be alleviated by inhibiting glycolysis in vitro. Treatment with AZD7545, a selective inhibitor of pyruvate dehydrogenase kinase, abrogated the increased expression of *Runx2*, *Mmp13*, *Adamts5*, and *Col10a1*, which are chondrocyte hypertrophy and catabolism markers, in Vhl-deficient cells. Chondrocyte hypertrophy has previously been linked to EP cartilage degeneration in DDD progression.^25^ Moreover, aberrant glycolytic metabolism causes hypertrophy-like changes in chondrocytes during OA development.^55^ Therefore, we hypothesized that the chondrocyte hypertrophy-like changes in EP might be related to the dysregulation of glycolytic metabolism in DDD. Our studies suggest that aberrant Hif1α signaling regulates chondrocyte hypertrophy and catabolism in part via activation of glycolytic metabolism. However, no significant changes in *Col2a1* or *Aggrecan* were observed following glycolysis inhibition, indicating that the Hif1α signaling-mediated anabolic effects on EP cartilage may be independent of glycolytic metabolism. Recent studies have shown the effects of stabilized HIF1α on osteoblast precursors, and it has been reported that stabilized HIF1α promotes bone formation via the activation of the glycolytic program.^33,47,56^ It is still not clear at present whether activated glycolysis accelerates the ossification of degenerative EP tissue in the IVD. It would be interesting to know whether inhibition of glycolysis will attenuate the EP ossification observed in DDD progression. To further genetically confirm the relationship between HIF1α and glycolysis signaling in IVD homeostasis, it is necessary to observe whether genetic inactivation of glycolysis, such as deletion of *Pdk1*, could rescue the DDD phenotypes observed in *Vhl cKO* mice.

Intraperitoneal (i.p.) administration of 2ME2, a pharmacological Hif1α inhibitor, attenuated mechanical stress-induced DDD pathogenesis, which further shows the harmful role of aberrantly enhanced HIF1α activity in DDD. Disc degeneration is not just a local disease confined to the spine, and some inflammatory mediators that regulate DDD pathogenesis are found in circulating blood.^57^ Inhibition of the HIF1α-related inflammatory response in other tissues/organs may also contribute to the therapeutic effects of i.p. administration of 2ME2 on DDD. One limitation of the current study is that the role of inflammatory cytokines in the DDD models was not explored. In addition, it is better to use *Vhl cKO* without TM injection as controls. In this study, considering the limited availability of *Vhl cKO* mice, we used floxed VHL mice without Col2-CreERT2 as a control, as we and others have done previously.^51,58^ To exclude the potential interference of *Col2-CreER*^*T2*^ in DDD pathogenesis, the discs of *Vhl cKO* without TM injection and Cre-negative mice were histologically analyzed using H&E staining at 12 months. There were no gross differences between Vhl cKO without TM and Cre-negative mice (Supplementary Fig 10S, T–U).

The identification of novel biotherapeutics based on mechanisms is essential for the clinical management of IVD degeneration. The findings of this study explored for the first time the role of aberrant HIF1α activation in DDD pathogenesis in vivo and further showed that inhibition of either aberrant HIF1α activation or HIF1α activation-mediated glycolysis can attenuate DDD pathology. These findings imply that modulating HIF1α signaling and/or glycolysis activity are potential therapeutic approaches for the management of disc degeneration.

## Materials and methods

### Animals

*Vhl*^*f/f*^ (*Vhl* floxed) mice and *Hif1α*^*f/f*^ (*Hif1α* floxed) mice were purchased from Jackson Laboratories (Bar Harbor, Maine, USA), and *Col2a1-CreER*^*T2*^ mice were generated in the laboratory of Prof. Di Chen. *Vhl*^*f/f*^ mice were crossed with *Col2a1-CreER*^*T2*^ mice to obtain *Vhl*^*f/f*^; *Col2a1-CreER*^*T2*^ (*Vhl* cKO) and *Vhl1*^*f/f*^ (Cre-negative) mice. *Vhl*^*f/f*^; *Col2a1-CreER*^*T2*^ mice were bred with *Hif1α*^*f/f*^ mice to obtain *Vhl*^*f/f*^ *Hif1α; Col2a1-CreER*^*T2*^ (*VhlHif1α* cKO) mice. *Col2a1-CreER*^*T2*^ transgenic mice were bred with *Rosa26* reporter mice. TM induction was performed in 2-month-old *Col2a1-CreER*^*T2*^*; R26R* mice. Mice were euthanized at the age of 4 months, and the Cre recombination efficiency was evaluated by Lac-Z staining.^54^ All mice were maintained on a C57BL/6J background. TM (Sigma-Aldrich, St. Louis, MO, USA) was administered by i.p. injection to *Vhl* cKO, *VhlHif1α* cKO, and Cre-negative male littermates at the age of 8 weeks old (1 mg per 10 g body weight, daily for 5 days). Mice were housed in SPF conditions at the animal facility of Daping Hospital (Chongqing, China).

### Looping tail mouse model

Tail-looping surgery was performed according to a protocol described previously.^59^ Briefly, 10-week-old C57BL/6J male mice were anesthetized, and the tails were looped at a fixed position between the 5th and 13th vertebrae using 0.8-mm stainless steel. After 1 week and 3 weeks, the mice were sacrificed, and the caudal vertebrae were harvested (*n* = 8 per group). C8-C10 were analyzed.

### Lumbar spine instability (LSI) mouse model

Eight-week-old male mice (C57BL/6J background) were anesthetized by pentobarbital sodium. LSI surgery was performed on the lumbar 3rd - lumbar 5th spinous processes by resecting the supraspinous and interspinous ligaments as previously described.^60^ Sham surgery was carried out by detaching the paravertebral muscles from the L3–L5 vertebrae only. Mice were euthanized at 2 and 4 weeks after the surgery (*n* = 6 per group).

### Human samples

Human EP and AF tissues were obtained from patients who underwent operations at Daping Hospital (Chongqing, China) (*n* = 9, modified Pfirrmann classification system grade 6–8, average age 64 years old). Normal human EP and AF tissues were obtained from patients with LVF (*n* = 3, modified Pfirrmann classification system grade 1–2, average age 26 years old). The sample details for the experiments are provided (SF1 A). Samples were collected with approval by the Institutional Review Board and Ethics Committee of Daping Hospital, and consent was obtained from the patients and families.

### Histological assessment

Mice were euthanized. Mouse spines were removed, cleaned of muscle and ligament, fixed in 4% paraformaldehyde, and stored at 4 °C. The specimens were decalcified in 10% EDTA (pH 7.0) for 3 to 4 days and embedded in paraffin and OCT compound (VWR) at –80 °C. Most analyses were performed on paraffin-embedded specimens, while detection of CD24, COLI, COX4, and CML was better in frozen specimens. For frozen tissue sections, slides were embedded in OCT compound (for 4-μm-thick slides) for 10 min at –20 °C. For paraffin sections, slides were dewaxed in xylene. Five-micrometer paraffin sections of the L4–L5 or C7–8 spines were processed for Safranin O/Fast green/Alcian blue (FAS) staining and H&E staining. For histomorphometric analysis, we used the EP degenerative histological score and NP-AF degenerative histological score to evaluate the whole IVD according to previous studies.^23,61^ Two blinded independent investigators evaluated each image and performed the scoring.

### X-ray analysis

X-ray images of whole IVD tissue were obtained using an MX-20 Cabinet X-ray system (Faxitron X-ray, Tucson, AZ, USA) according to our previous study.^62^

### 2-Methoxyestradiol (2ME2) treatment

Wild-type C57BL/6 mice were subjected to LSI or sham surgery at 8 weeks old, immediately followed by injection of Hif1α inhibitor (2ME2, EMD Millipore Corporation).^47^ 2ME2 was administered at 75 mg·kg^−1^ via i.p. injection for 2 weeks or 4 weeks. Vehicle groups were treated with solvent in equivalent volumes. We harvested lumbar spine samples at 2 and 4 weeks after LSI surgery (*n* = 6 per group). For the dosage-screening experiments, 2-month-old sham-operated and LSI mice were assigned into 5 groups with 4–5 mice per group. Three days after LSI surgery, mice were injected with several doses (50, 75, and 100 mg·kg^−1^) of Hif1α inhibitor (2ME2) or the equivalent volume of vehicle intraperitoneally (*n* = 5 per group). The spine samples were harvested at 4 weeks after LSI surgery.

### Immunofluorescence staining and confocal laser scanning

Slides were rinsed with PBS and incubated with blocking buffer (1% BSA in PBS) for 30 min. Sections were incubated overnight at 4 °C with antibodies against the following proteins: CD24 (1:200 dilution; Abcam, MA, USA), type I collagen (1:200 dilution; Abcam, MA, USA), CML (1:100 dilution; Abcam, MA, USA), and COX4 (1:200 dilution; Abcam, MA, USA). After being washed three times with PBS, slides were incubated with secondary antibodies. The IVD tissues were recorded using a confocal microscope (Zeiss LSM880, Germany).

### Assays of glucose consumption and lactate production

A microplate reader system (Thermo Scientific, Varioskan Lux) was used to measure glucose consumption and lactate production. Cells were plated in a 96-well plate at a density of 10 000 cells/well and incubated in a 37 °C incubator for 24 h. After the indicated treatment, cells were incubated with glucose consumption and lactate production reagents according to the manufacturer’s recommendations (BC2500 for glucose consumption assay and BC2230 for lactate production assay, Solarbio, China). The assay signals of glucose consumption and lactate production were collected at 5-min intervals for approximately 120 min using excitation and emission wavelengths of 380 and 615 nm, respectively.

### Measurement of mitochondrial membrane potential (ΔΨm)

We performed JC-1 (Beyotime Biotech, China) assays to assess the mitochondrial membrane potential (△Ψm) according to the manufacturer’s instructions. Briefly, the cells were incubated at 37 °C for 1 h with JC-1 (5 mg·L^−1^), rinsed twice with PBS and placed in fresh medium without serum. Images were acquired at 490 excitation and 530 emission for green and at 540 excitation and 590 emission for red using a confocal microscope (Zeiss LSM880, Germany).

### Measurement of mitochondrial mass

We performed a MitoTracker^®^ (Invitrogen, USA) assay to observe mitochondrial morphology. When cells reached the desired confluency (70%–80%), the media was removed from the dish, prewarmed (37 °C) staining solution containing MitoTracker® probe (200 nmol·L^−1^) was added, and the cells were incubated for 30 min under growth conditions. After staining was completed, the staining solution was replaced with fresh prewarmed media or buffer, and the cells were observed using a fluorescence microscope (490 excitation and 510 emission) (Zeiss LSM880, Germany). For quantification of mitochondrial mass, cells were harvested by trypsinization and washed twice in PBS. Cells were then resuspended in 100 μL of PBS containing 200 nmol·L^−1^ MitoTracker Green and incubated at 37 °C for 45 min. Cells were then washed twice in PBS and analyzed by flow cytometry in a flow cytometer using a 488 nm argon excitation laser.

### Measurement of ROS generation

We performed a DHE (Thermo Fisher) assay to assess the ROS levels according to the manufacturer’s instructions. Briefly, culture medium was removed, and cells were washed once with DPBS and incubated in fresh culture medium without FBS. The cells were then stained with 5 μmol·L^−1^ DHE Green Reagent and Hoechst 33342 by adding the probe to the complete media and incubating at 37 °C for 30 min. Fluorescence images were obtained at 485 excitation and 520 emission. The fluorescence intensity values from three different fields of view were determined using Image-Pro Plus 5.1 (Media Cybernetics, Rockville, MD, USA), and the mean values were calculated.

### Immunohistochemistry

Immunohistochemistry was performed using SP-9000 Histostain-Plus kits (ZSGB-BIO, Beijing, China) according to a previous study.^63^ Briefly, decalcified IVD tissues were deparaffinized with xylene, and endogenous peroxidase activity was quenched with 3% H_2_O_2_. Antigen retrieval was performed with 0.1% trypsin, and normal goat serum was used for blocking for 30 min. Sections were incubated overnight with anti-Hif1α (1:200 dilution; Abcam, MA, USA), anti-Hif2α (1:200 dilution; Abcam, MA, USA), anti-VHL (1:200 dilution; Abcam, MA, USA), type X collagen (COLX) (1:400 dilution; Abcam, MA, USA), anti-osteocalcin (1:100 dilution; Santa Cruz Biotechnology), anti-RUNX2 (1:100 dilution; Santa Cruz Biotechnology), anti-VEGF (1:200 dilution; Abcam, MA, USA), anti-GLUT1 (1:200 dilution; Millipore, Billerica, MA, USA), anti-LDHA (1:200 dilution; Abcam, MA, USA), and anti-PDK1 (1:200 dilution; Abcam, MA, USA). After rinsing with PBS, a horseradish peroxidase (HRP)-conjugated secondary antibody was applied and stained with a DAB kit.

### Western blotting

Equal amounts of protein samples were resolved on a 10%–12% SDS-PAGE gel and transferred onto a polyvinylidene difluoride membrane. After blocking with 5% nonfat milk, the membrane was probed with primary antibodies specific for GLUT1 (Abcam), PDK1 (Abcam), and VHL (Abcam) followed by secondary antibodies, and the signal was detected with chemiluminescence (Pierce) according to the manufacturer’s protocols. β-Actin (Sigma-Aldrich) was applied to normalize the protein expression levels.

Primary chondrocytes from mice were collected using a surgical microscope as described previously.^64,65^ Primary chondrocytes were digested with 0.2% collagenase II (Sigma-Aldrich) in DMEM/F12 medium (HyClone) for 12 h at 37 °C. The suspended cells were then filtered through a 70-mm cell filter. The suspension was centrifuged for 5 min at 1 000 r·min^−1^. After removing the suspension solution, the pellet was resuspended in a culture medium containing DMEM/F12, 10% FCS, and 1% penicillin-streptomycin (HyClone). Then, the primary chondrocytes were washed twice with PBS, and cell lysis buffer with protease inhibitor was added to the culture dish. The protein lysates were extracted into EP tubes for western blotting.

### Cell line cultures and treatments

iAF cells (provided by Prof. Di Chen, Chinese Academy of Sciences, China) were cultured in DMEM/F12 (1:1) supplemented with 20% FBS and Y27632 (MFCD03490488, Merck, USA). RCS cells (provided by Dr. Yeguang Chen, Tsinghua University, China) were cultured in DMEM/F12 (1:1) supplemented with 5% FBS. Transient transfection (siRNA^vhl^) was performed with VigoFect (T001, Vigorous) in accordance with the manufacturer’s protocols. The RCS cell lines were treated with 10 μmol·L^−1^ PDK1 inhibitor (ADZ7545; Selleck Chemicals) or vehicle (dimethyl sulfoxide; Sigma-Aldrich) for 24 h.

### Real-time PCR

Total RNA was isolated using TRIzol reagent (Invitrogen, M7514, USA). Real-time PCR was performed using a Max3000 PCR machine (Stratagene, USA) and SYBR Premix Ex TaqTM kit (Takara, RR047A) at least three times. mRNA expression was normalized to the corresponding values of GAPDH. The sequences for the primers used were as follows: *Ppargc1a* 5′-TATGGAGTGACATAGAGTGTGCT-3′ and 5′-CCACTTCAATCCACCCAGAAAG-3′; *Mxi* 5′-AACATGGCTACGCCTCATCG-3′ and 5′-CGGTTCTTTTCCAACTCATTGTG-3′; *Mfn1* 5′-CCTACTGCTCCTTCTAACCCA-3′ and 5′-AGGGACGCCAATCCTGTGA-3′; *Mfn2* 5′-CCTACTGCTCCTTCTAACCCA-3′ and 5′-AGGGACGCCAATCCTGTGA-3′; *Opa1* 5′-TGGAAAATGGTTCGAGAGTCAG-3′ and 5′-CATTCCGTCTCTAGGTTAAAGCG-3′; *Ant1* 5′-GAGGCGTGGATCGCCATAAG-3′ and 5′-CACTTGGGGGAGTTCATGCT-3′; *CypD* 5’-CTTCCACAGGGTGATCCCAG-3’ and 5′-ACTGAGAGCCATTGGTGTTGG-3′; *Ucp3* 5′-CTGCACCGCCAGATGAGTTT-3′ and 5′-ATCATGGCTTGAAATCGGACC-3′; *Mmp13* 5′-CTTTGGCTTAGAGGTGACTGG-3′ and 5′-AGGCACTCCACATCTTGGTTT-3′; *Admats5* 5′-CGCTACACTCTAAAGCCACTC-3′ and 5′-CCTCGAAGCTAAAGCCCTCG-3′; *Pdk1* 5′-GCACTCCTTATTGTTCGGTGG-3′ and 5′-CGTCGCAGTTTGGATTTATGCT-3′; *Runx2* 5′-AGAGTCAGATTACAGATCCCAGG-3′ and 5′-TGGCTCTTCTTACTGAGAGAGG-3′; *Col2al* 5′-CCTGGACCCCGTGGCAGAGA-3′ and 5′-CAGCCATCTGGGCTGCAAAG-3′; *Col10al* 5′-GGGTAAAGAGATTTCAGTAAGAGGA-3′ and 5′-GTCCAGGACTTCCATAGCCT-3′; *Aggrecan* 5′-GGGACCCCAAGGACCTAAAG-3′ and 5′-GCCCAACTAGACCTATCTCACCT-3′; *GAPDH* 5′-CCACAGTCCATGCCATCAC-3′ and 5′-TCCACCACCCTGTTGCTGTA-3′.

For human samples, total RNA was extracted from human EP tissues using QIAzol Lysis Reagent (Qiagen; CA; USA) and the miRNeasy Mini Kit (Qiagen; CA; USA). The total amount of isolated RNA was retrotranscribed to cDNA using the PrimeScript ® Reverse Transcription kit (Takara Biomedical Technology; Beijing; China). After the enzyme reaction, the cDNA concentration was measured and adjusted to 250 ng·μL^−1^. mRNA expression was normalized to the corresponding values of ACTB. The sequences for the primers used were as follows: *HIF1A* 5′-GAACGTCGAAAAGAAAAGTCTCG-3′ and 5′-CCTTATCAAGATGCGAACTCACA-3′; *HIF2A* 5′-CGGAGGTGTTCTATGAGCTGG-3′ and 5′-AGCTTGTGTGTTCGCAGGAA-3′; *HIF3A* 5′-GCACCCTCAACCTCAAGGC-3′ and 5′-GCAATCCTGTCGTCACAGTAG-3′; *ACTB* 5′-CATGTACGTTGCTATCCAGGC-3′ and 5′-CTCCTTAATGTCACGCACGAT-3′.

### Statistical analysis

All numeric data are presented as the mean ± SD. Error bars indicate SD. Differences between two groups were evaluated using unpaired Student’s *t* test, and one-way ANOVA was used for comparisons of multiple groups (3 or more groups). All statistical analyses were performed using GraphPad PRISM 7.0 software, and *P* < 0.05 was considered statistically significant.

### Study approval

All in vivo experiments and protocols were approved by the Institutional Animal Care and Use Committee of the Research Institute of Surgery, Daping Hospital, IACUC protocol SCXK- (Army) 2007–017.

## Supplementary information

Supplementary Figure1

Supplementary Figure2

Supplementary Figure3

Supplementary Figure4

Supplementary Figure5

Supplementary Figure6

Supplementary Figure7

Supplementary Figure8

Supplementary Figure9

Supplementary Figure10

Supplemental Fig legends
