# Supplementary figures and images for: Inhibition of aberrant Hif1α activation delays intervertebral disc degeneration in adult mice
Source: Bone Res. 2022 Jan 5;10:2. doi: 10.1038/s41413-021-00165-x (PMC8727577; doi:10.1038/s41413-021-00165-x)

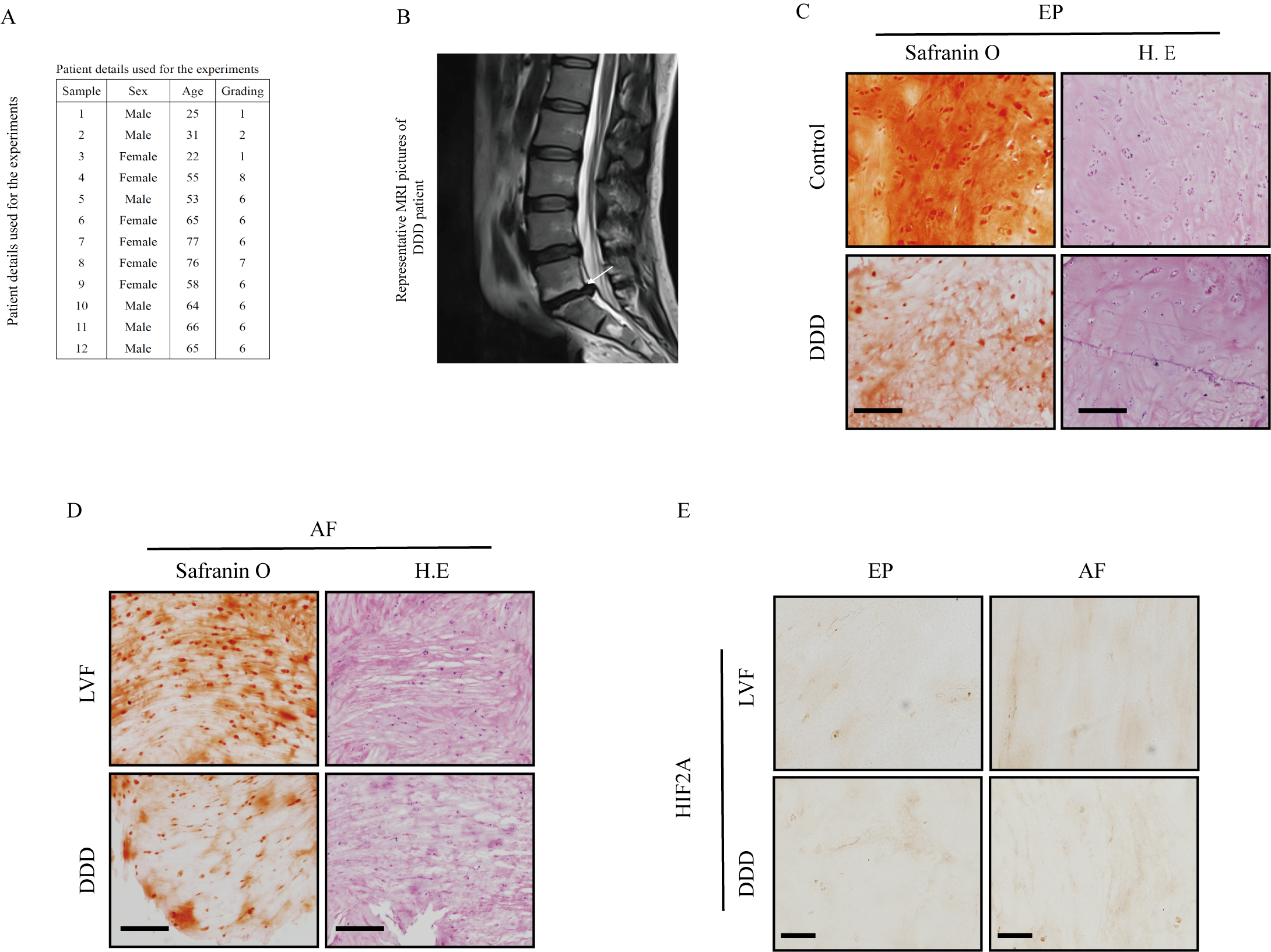

Supplement: Supplementary file 1 — Supplementary Figure1 [file 41413_2021_165_MOESM1_ESM.tif]

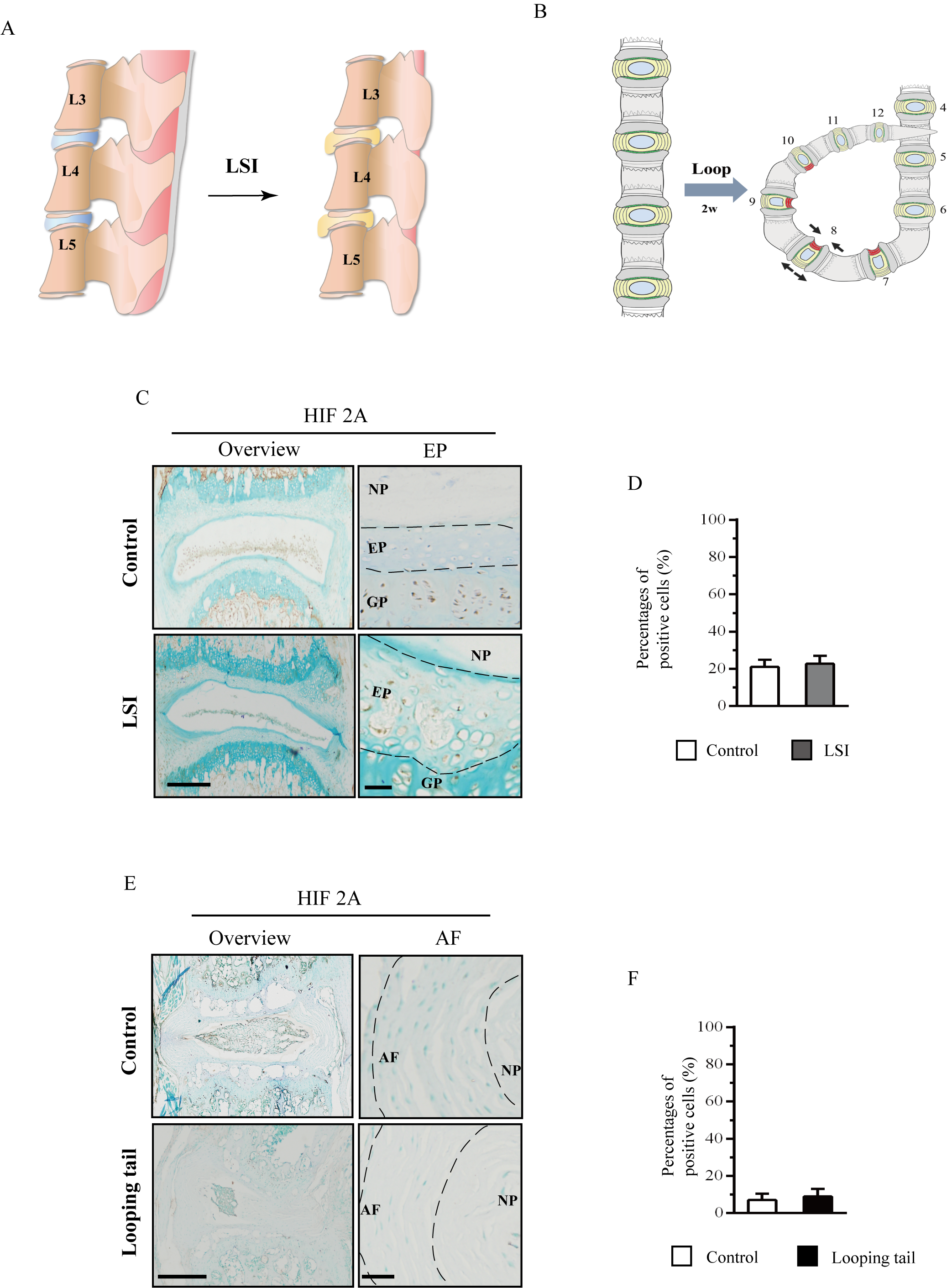

Supplement: Supplementary file 2 — Supplementary Figure2 [file 41413_2021_165_MOESM2_ESM.tif]

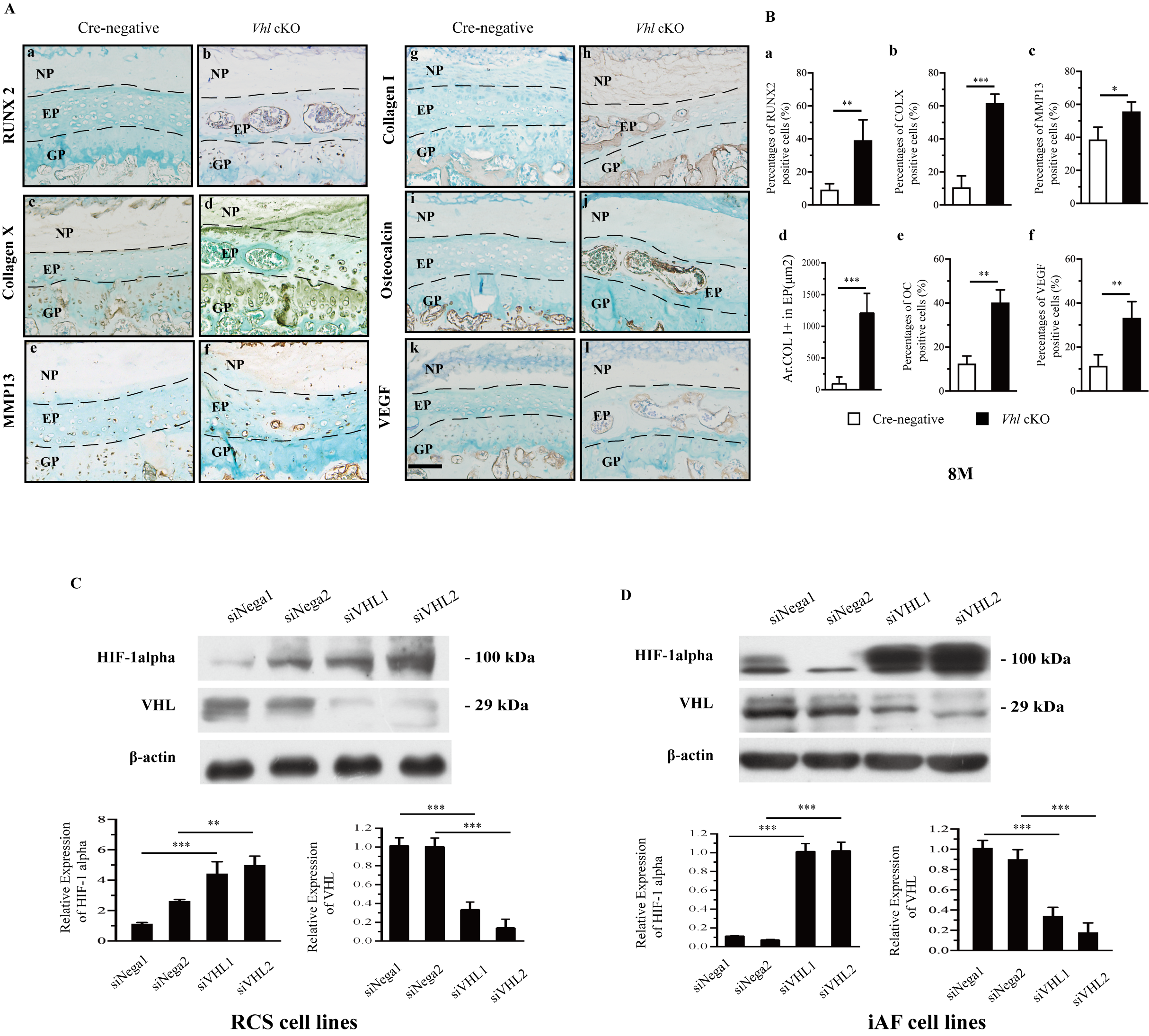

Supplement: Supplementary file 3 — Supplementary Figure3 [file 41413_2021_165_MOESM3_ESM.tif]

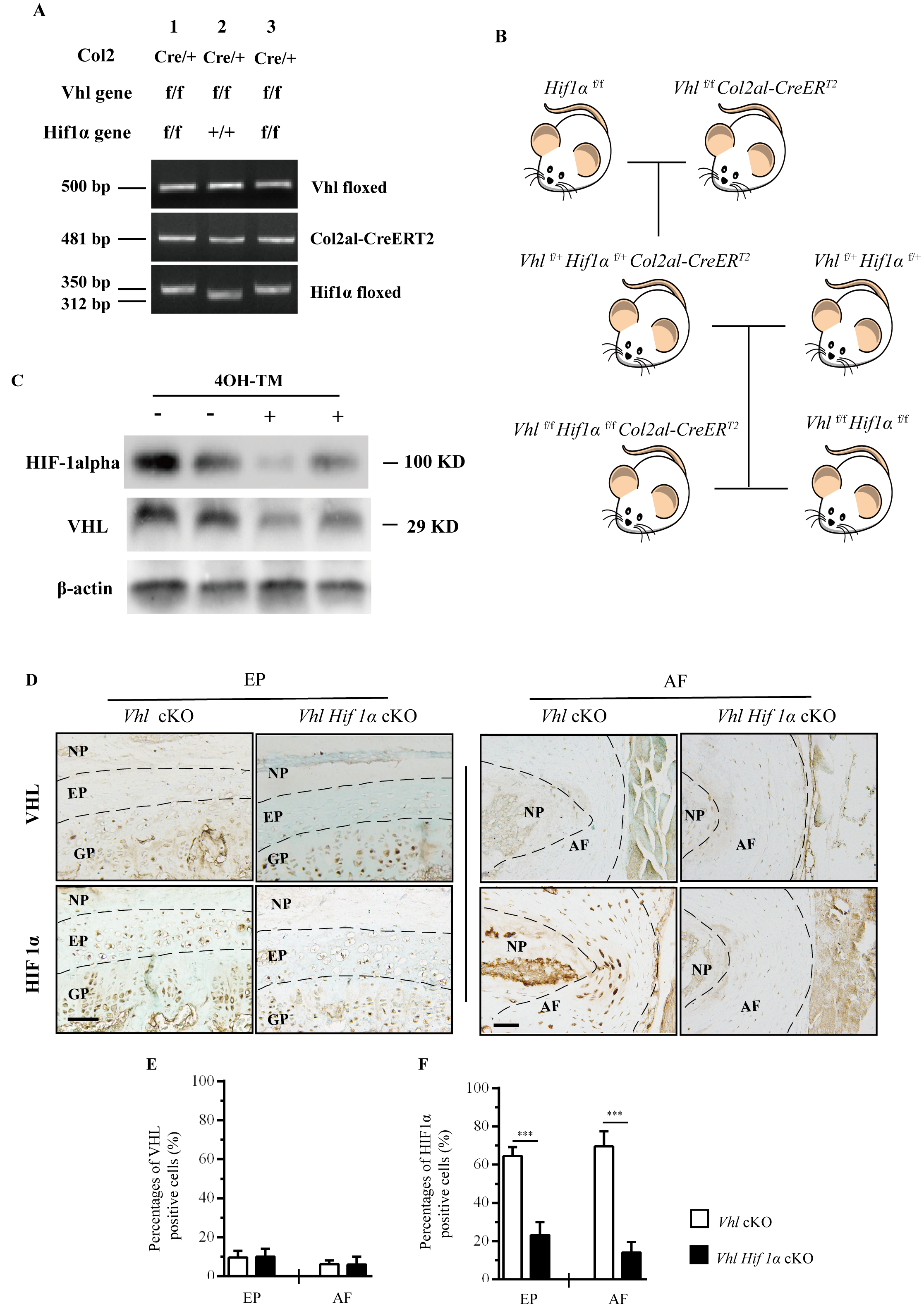

Supplement: Supplementary file 4 — Supplementary Figure4 [file 41413_2021_165_MOESM4_ESM.tif]

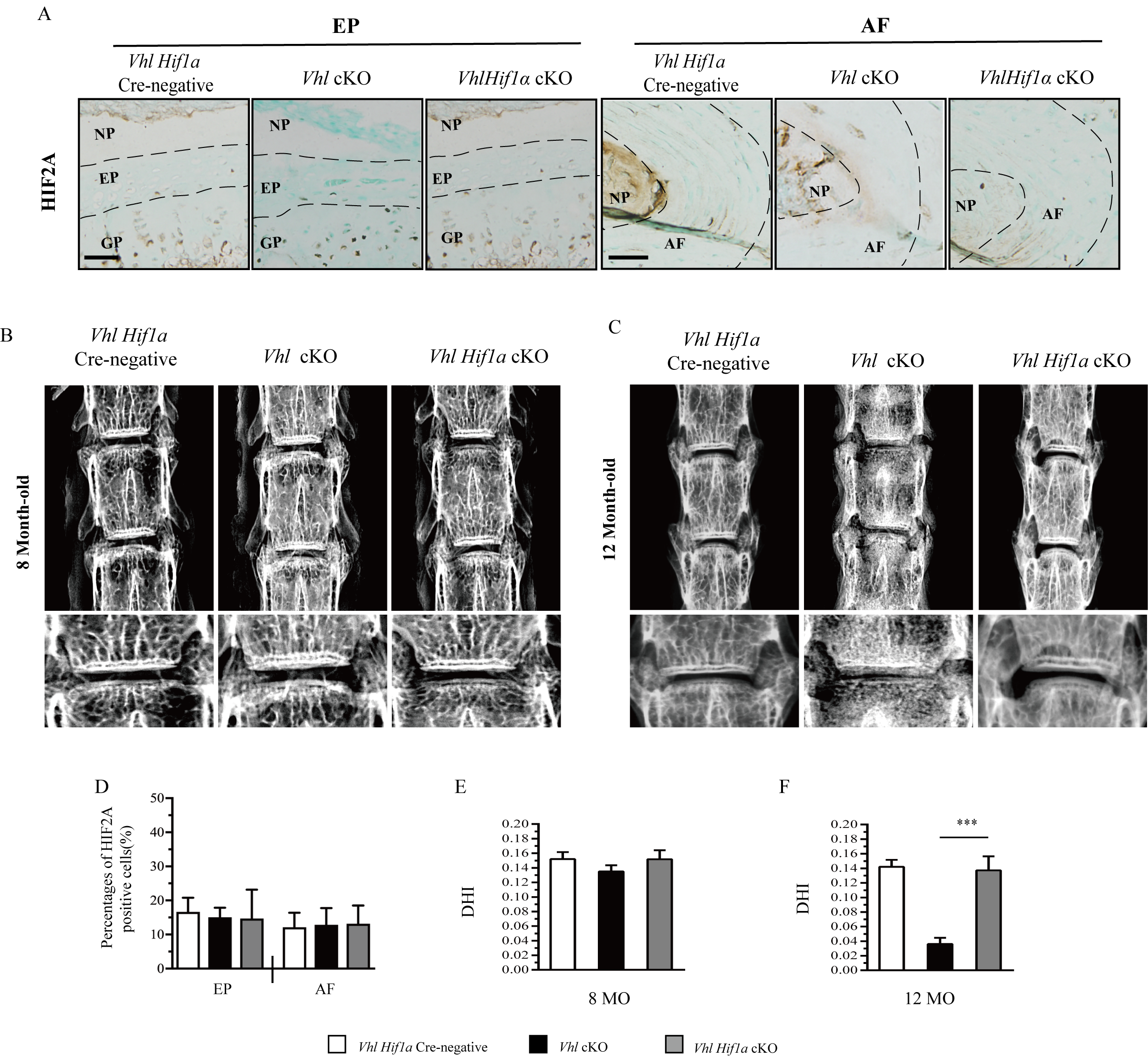

Supplement: Supplementary file 5 — Supplementary Figure5 [file 41413_2021_165_MOESM5_ESM.tif]

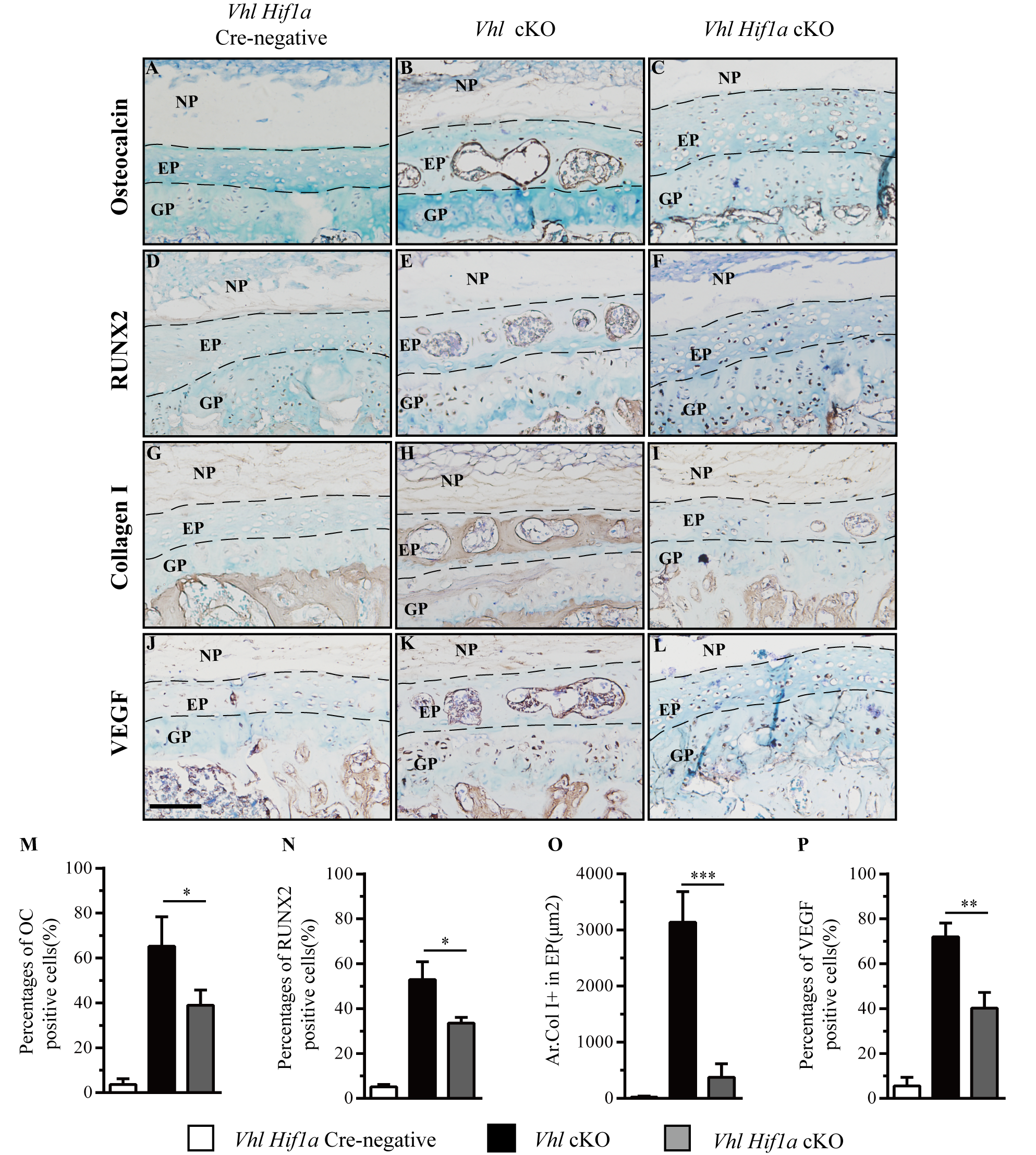

Supplement: Supplementary file 6 — Supplementary Figure6 [file 41413_2021_165_MOESM6_ESM.tif]

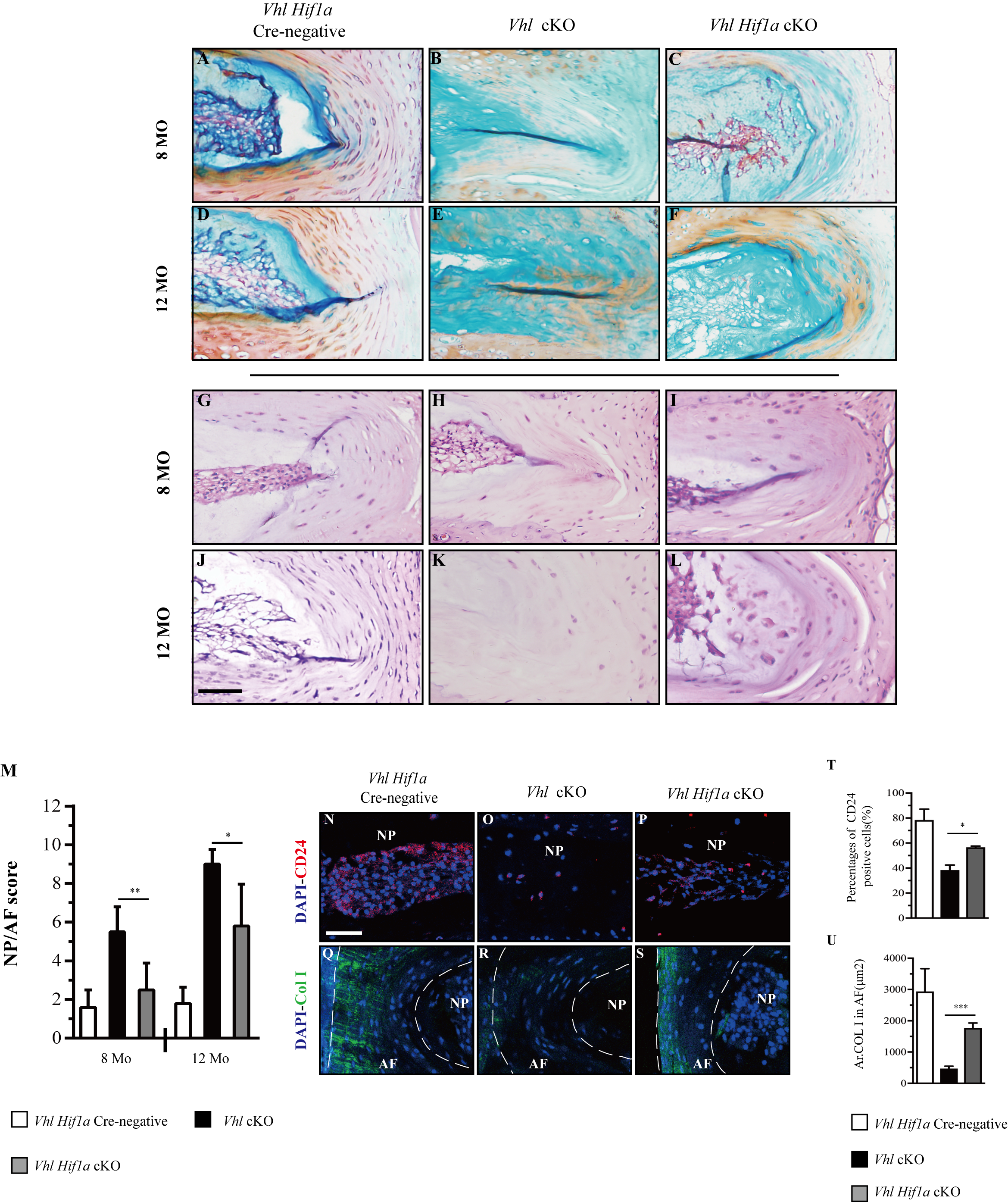

Supplement: Supplementary file 7 — Supplementary Figure7 [file 41413_2021_165_MOESM7_ESM.tif]

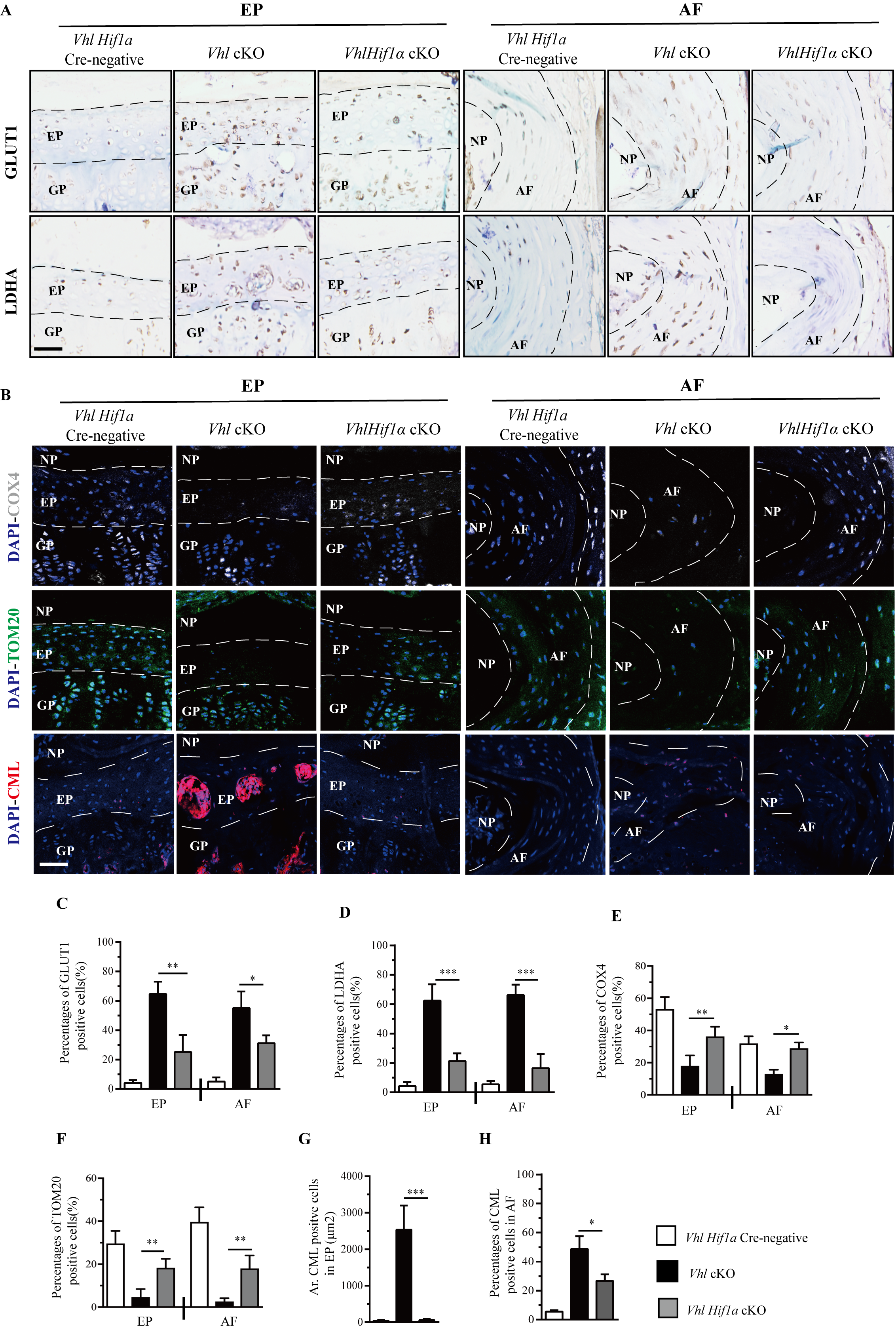

Supplement: Supplementary file 8 — Supplementary Figure8 [file 41413_2021_165_MOESM8_ESM.tif]

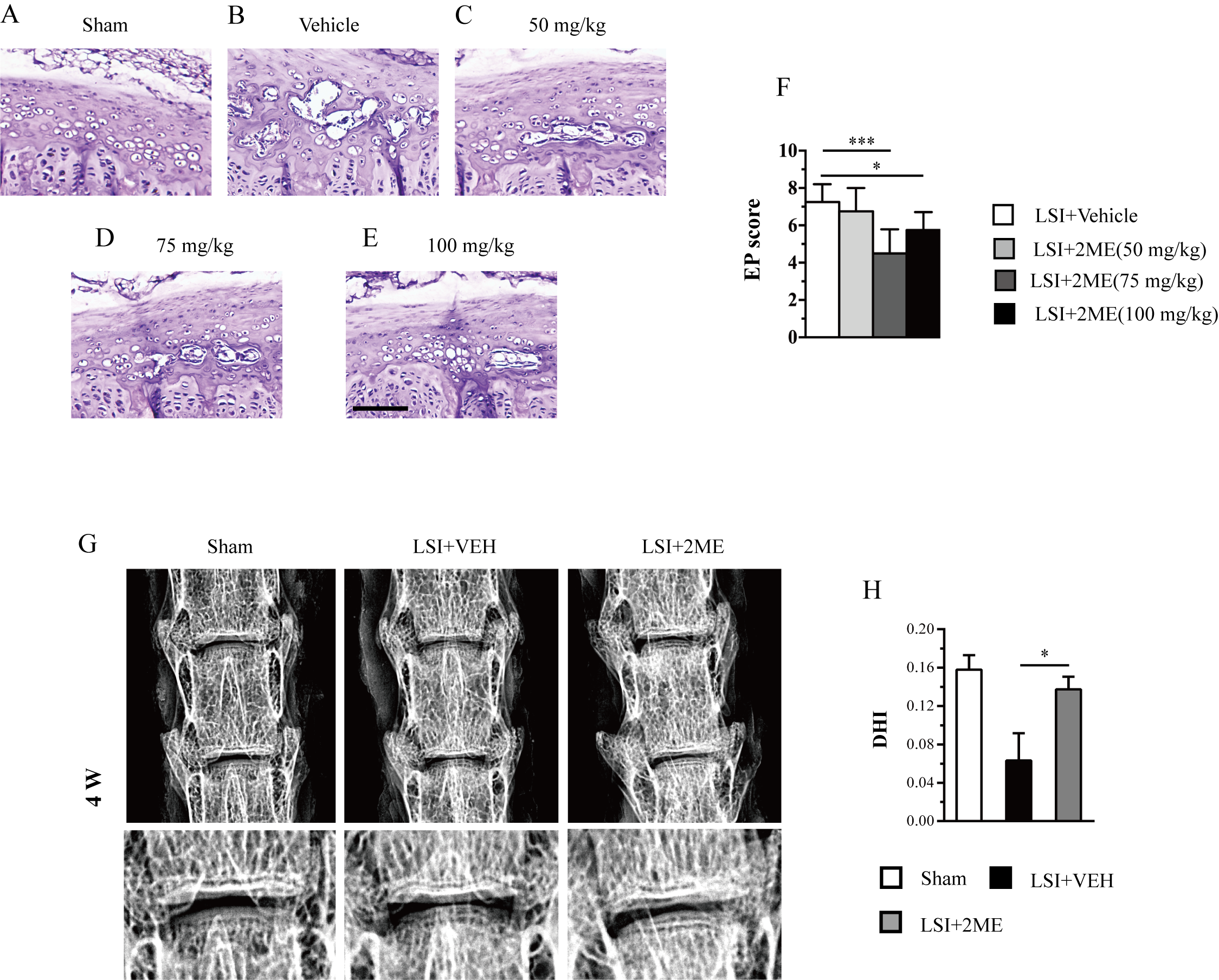

Supplement: Supplementary file 9 — Supplementary Figure9 [file 41413_2021_165_MOESM9_ESM.tif]

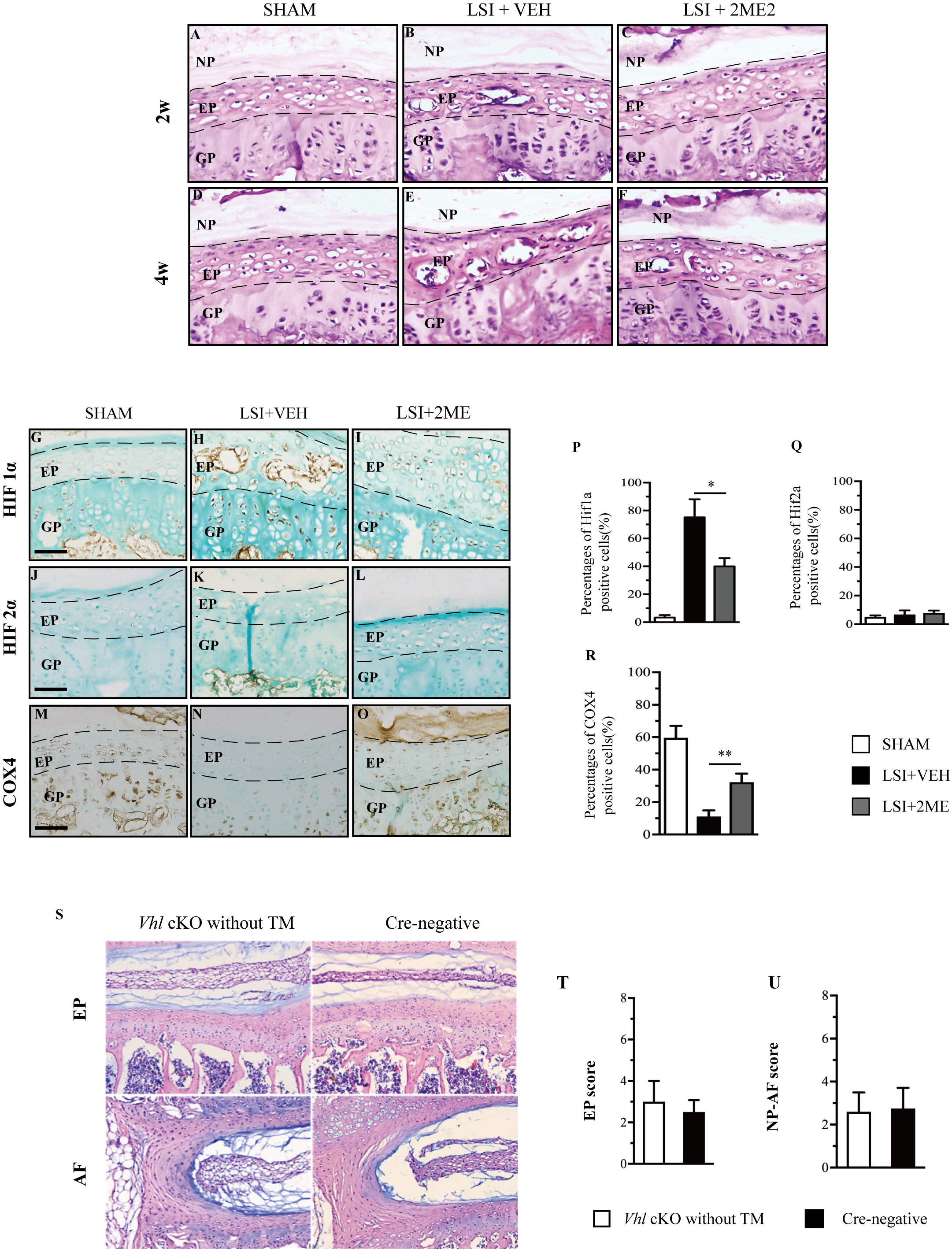

Supplement: Supplementary file 10 — Supplementary Figure10 [file 41413_2021_165_MOESM10_ESM.tif]
