# Supplementary material for: Inhibition of aberrant Hif1α activation delays intervertebral disc degeneration in adult mice
Source: Bone Res. 2022 Jan 5;10:2. doi: 10.1038/s41413-021-00165-x (PMC8727577; doi:10.1038/s41413-021-00165-x)
Supplement: Supplementary file 11 — Supplemental Fig legends [file 41413_2021_165_MOESM11_ESM.docx]

**Supplementary Figure 1.** (A) Patients’ information for the experiments. (B) Representative MRI pictures from the patients with DDD. (C-D) Representative fast green/safranin O- and H & E-stained images of normal and degenerative human lumbar EP and AF tissues. (E) Immunohistochemical detection of HIF2A expression was performed in normal and degenerative lumbar EP and AF tissues. Scale bar: 200µm(C-E).

**Supplementary Figure 2.** (A-B) Schematic diagram of two experimental mouse models resulting from aberrant mechanical loading. (C) IHC staining for HIF2α was performed in IVD of wild-type mice 2 weeks after LSI surgery. (D) Quantitative analysis of the areas of HIF2α immunoreacted positive cells in LSI surgery. (E) Immunohistochemical detection of HIF2α expression was performed 1 weeks after tail-looping surgery. (F) Quantitative analysis of HIF2α+ cells. Data are expressed as the percent expression relative to controls. Values represent mean (symbols) ± SD (error bar). P-values between groups with * are less than 0.05.

**Supplementary Figure 3.** (A) Immunostaining and (B) quantitative analysis of RUNX2^+^ cells COLX^+^ cells, MMP13^+^ cells, COLI^+^ cells, OC+ cells and VEGF^+^ cells in lumbar discs from WT, *Vhl* cKO mice at the age of 8 months (n=3 per group). Scale bar: 50µm(A). (C) Western blot analysis of the HIF1α and VHL expression in RCS cell line with siRNA-Vhl, siNega group were used as controls. (D) Western blot analysis of the HIF1α and VHL expression in iAF cell line with siRNA-Vhl, siNega group were used as controls. Data are expressed as the percent expression relative to controls. Values represent mean (symbols) ± SD (error bar). P-values between groups with * are less than 0.05.

**Supplementary Figure 4.** (A) Polymerase chain reaction (PCR) for genotyping of *Vhl^flox/flox^ Hif1α^flox/flox^Col2a1-Cre^ERT2^* (*VhlHif1α* cKO) mice. (B) Scheme of experiment: *Hif1α*^f/f^ mice were crossed with *Vhl^flox/flox^Col2a1-Cre^ERT2^* mice to generate *Vhl^flox/flox^ Hif1α^flox/flox^Col2a1-Cre^ERT2^* and control mice. (C) Western blot analysis of the VHL and HIF1-alpha expressions in the primary IVD cells of *VhlHif1α* cKO with tamoxifen (TM) treatment. (D) Immunohistochemistry analysis of VHL and HIF1α in the EP and AF of *Vhl^flox/flox^ Hif1α^flox/flox^Col2a1-Cre^ERT2^* and control mice (n=4 per group). (E-F) Quantitative analysis of VHL+ cells and HIF1α+ cells in EP and AF of *VhlHif1α* cKO and control mice. Scale bar: 50µm(D). Data are expressed as the percent expression relative to controls. Values represent mean (symbols) ± SD (error bar). P-values between groups with * are less than 0.05.

**Supplementary Figure 5.** (A) Immunohistochemistry analysis of HIF2α in the EP and AF of *Vhl^flox/flox^ Hif1α^flox/flox^Col2a1-Cre^ERT2^* and control mice (n=4 per group). (B-C) Radiographic assessment of IVD phenotypes in *VhlHif1α* cKO mice and control mice at 8 and 12 months of age. (D) Quantitative analysis of HIF2α+ cells in EP and AF of *VhlHif1α* cKO and control mice. Scale bar: 50µm(D). (E-F) Quantitative analysis of height index (DHI). Data are expressed as the percent expression relative to controls. Values represent mean (symbols) ± SD (error bar). P-values between groups with * are less than 0.05.

**Supplementary Figure 6.** (A-L) Immunostaining and (M-P) quantitative analysis of Osteocalcin^+^ cells, RUNX2^+^ cells, COLI^+^ cells and VEGF + cells in lumbar discs from WT, *Vhl* cKO and *Vhl Hif1α cKO* mice (n=3 per group). Scale bar: 50µm(A-E). Data are expressed as the percent expression relative to controls. Values represent mean (symbols) ± SD (error bar). P-values between groups with * are less than 0.05.

**Supplementary Figure 7.** (A-L) Fast Green/Safranin O- and H & E-stained coronal sections of the AF and NP from WT, *Vhl* cKO and *Vhl Hif1α cKO* mice (n=5-6 per group). (M) Degenerative scores of NP/AF tissues of WT, *Vhl* cKO and *Vhl Hif1α cKO* mice. (N-P) CD24 signals were analyzed by immunofluorescence assay from NP cells of WT, *Vhl* cKO and *Vhl Hif1α cKO* mice at age 12months. (red; anti-CD24; blue; Hoechst) (n=3-4 per group). (Q-S) Representative COL I immunofluorescence images of IVDs from lumbar disc AF of 12-month-old WT, *Vhl* cKO and *Vhl Hif1α cKO* mice (green; anti-Col I; blue; Hoechst) (n=3-4). (T-U) Quantitative analysis of COL I + cells and CD24+ cells in EP and AF of WT, *Vhl* cKO and *Vhl Hif1α cKO* mice. Scale bar: 50µm(A-E). Data are expressed as the percent expression relative to controls. Values represent mean (symbols) ± SD (error bar). P-values between groups with * are less than 0.05.

**Supplementary Figure 8.** (A-B) Immunostaining and (C-H) quantitative analysis of GLUT1^+^ cells, LDHA^+^ cells, COX4^+^ cells (white; anti-COX4; blue; Hoechst), TOM20^+^ cells (green; anti-TOM20; blue; Hoechst) and CML^+^ cells (red; anti-CML; blue; Hoechst) in EP and AF tissues from WT, *Vhl* cKO and *Vhl Hif1α cKO* mice (n=3 per group). Scale bar: 50µm(A-B). Data are expressed as the percent expression relative to controls. Values represent mean (symbols) ± SD (error bar). P-values between groups with * are less than 0.05.

**Supplementary Figure 9.** (A-E) Screen of the optimal dose of 2ME for the treatment of LSI mice. (F) Histological degenerative scores of EP cartilage in LSI-surgery mice. (G) Radiographic assessment of IVD phenotypes in mice with or without LSI surgery. (H) Quantitative analysis of height index (DHI). Scale bar: 50µm(A-E). Data are expressed as the percent expression relative to controls. Values represent mean (symbols) ± SD (error bar). P-values between groups with * are less than 0.05.

**Supplementary Figure 10.** (A-F) IVD samples were harvested at 2- and 4-weeks post-LSI surgery and analyzed histologically by H＆E staining. (G-O) HIF1α, HIF2α and COX4 signals were analyzed by immunofluorescence assay from IVDs of mice with or without LSI surgery and (P-R) percentages of HIF1α, HIF2α and COX4 immunoreacted cells was quantified (n =4 per group). (S)H. E staining of lumbar IVDs of *Vhl* cKO mice without TM and Cre-negative mice at 12 months (n=7-8). (T-U) Histological degenerative scores in *Vhl* cKO mice without TM and Cre-negative mice. Scale bar: 50µm (A-F, G-O), 100µm (S). Data are expressed as the percent expression relative to controls. Values represent mean (symbols) ± SD (error bar). P-values between groups with * are less than 0.05.
